# Shifts in the functional capacity and metabolite composition of the gut microbiome during recovery from enteric infection

**DOI:** 10.3389/fcimb.2024.1359576

**Published:** 2024-05-08

**Authors:** Zoe A. Hansen, Anthony L. Schilmiller, Douglas V. Guzior, James T. Rudrik, Robert A. Quinn, Karla A. Vasco, Shannon D. Manning

**Affiliations:** ^1^ Department of Microbiology, Genetics, and Immunology, Michigan State University E., Lansing, MI, United States; ^2^ Research Technology Support Facility, Mass Spectrometry and Metabolomics Core, Michigan State University E., Lansing, MI, United States; ^3^ Department of Biochemistry and Molecular Biology, Michigan State University E., Lansing, MI, United States; ^4^ Michigan Department of Health and Human Services, Bureau of Laboratories, Lansing, MI, United States

**Keywords:** metabolomics, gut microbiome, enteric infection, diarrhea, metagenomics

## Abstract

While enteric pathogens have been widely studied for their roles in causing foodborne infection, their impacts on the gut microbial community have yet to be fully characterized. Previous work has identified notable changes in the gut microbiome related to pathogen invasion, both taxonomically and genetically. Characterization of the metabolic landscape during and after enteric infection, however, has not been explored. Consequently, we investigated the metabolome of paired stools recovered from 60 patients (cases) during and after recovery from enteric bacterial infections (follow-ups). Shotgun metagenomics was applied to predict functional microbial pathways combined with untargeted metametabolomics classified by Liquid Chromatography Mass Spectrometry. Notably, cases had a greater overall metabolic capacity with significantly higher pathway richness and evenness relative to the follow-ups (p<0.05). Metabolic pathways related to central carbon metabolism, amino acid metabolism, and lipid and fatty acid biosynthesis were more highly represented in cases and distinct signatures for menaquinone production were detected. By contrast, the follow-up samples had a more diverse metabolic landscape with enhanced richness of polar metabolites (p<0.0001) and significantly greater richness, evenness, and overall diversity of nonpolar metabolites (p<0.0001). Although many metabolites could not be annotated with existing databases, a marked increase in certain clusters of metabolites was observed in the follow-up samples when compared to the case samples and vice versa. These findings suggest the importance of key metabolites in gut health and recovery and enhance understanding of metabolic fluctuations during enteric infections.

## 1 Introduction

Microbes in the human gut have been shown to contribute to host metabolic health by breaking down complex carbohydrates and converting compounds into forms usable by the body (Oliphant and Allen-Vercoe, 2019). Specifically, these microorganisms generate beneficial short-chain fatty acids (SCFAs) like butyrate, acetate, and propionate that play crucial roles in counteracting inflammation and immune disorders (Richards et al., 2016). Successful production of these compounds, however, relies on specific microbes, most commonly those in the Bacteroidetes and Firmicutes phyla, whose abundance can change with diet and other perturbations (Wolters et al., 2019; Fusco et al., 2023).

Gut microbial composition has been linked to various disease states and the impacts of disease on the human gut microbiome and metabolome have been characterized using multi-omics approaches (Fan and Pedersen, 2021). Defining a “healthy gut” has been difficult due to the high level of variation in microbial and metabolic profiles across and within individuals. Indeed, changes in factors such as diet, exercise, nutrient load, age, and colonic transit time can impact the gut microbiome (Jumpertz et al., 2011; Roager et al., 2016; Singh and Manning, 2016; Rothschild et al., 2018). Because host metabolic health is strongly influenced by the gut microbiota, less diverse microbiomes have been linked to enhanced risk of metabolism-related diseases (Le Chatelier et al., 2013). Genetic variation among members of the same microbial taxa can also lead to differences in metabolic capacity and health (Zeevi et al., 2019). The interplay between the fluctuating human gut microbiome and related metabolic consequences in the context of disease, however, is understudied.

Numerous studies have explored microbial differences between otherwise healthy individuals and those with autoimmune and autoinflammatory disorders such as inflammatory bowel disease (IBD), and metabolic disorders like obesity and diabetes (Schippa and Conte, 2014). Obesity, for example, was associated with an increased Firmicutes : Bacteroidetes ratio and lower overall bacterial diversity (Candido et al., 2018), while prolonged consumption of a low-fiber diet was linked to the extinction of beneficial microbes (Sonnenburg et al., 2016). Furthermore, IBD patients had different gut-derived bile acids than healthy individuals, which can influence the host response to intestinal inflammation (Duboc et al., 2012). Understanding the relationship between the gut microbiota and metabolism in the presence of inflammation is of great relevance for many conditions and disorders.

Of particular interest to this study are metabolic shifts in the gut caused by foodborne bacterial pathogens, which were linked to 25,866 cases of infections, 6,164 hospitalizations, and 122 deaths in the United States in 2019 (Tack et al., 2020). Few studies, however, have explored the impact of acute enteric bacterial infections on the metabolic composition of the gut. In our prior studies, we documented shifts in taxonomic and resistance gene composition and diversity in patients with enteric bacterial infections relative to their healthy family members using 16S rRNA sequencing and metagenomics (Singh et al., 2015; Hansen et al., 2021, 2023). We therefore hypothesized that these shifts would also contribute to significant alterations in the human gut metabolome. To test this hypothesis, we characterized the metabolic trajectories in the gut among 60 patients during and after enteric infection using metagenome analysis and untargeted metabolomics.

## 2 Materials and methods

### 2.1 Study population and sample collection

We conducted a longitudinal case-control study in Michigan in collaboration with the Michigan Department of Health and Human Services (MDHHS) between 2011 and 2015 as described (Singh et al., 2015; Hansen et al., 2023). Sixty-one stool samples were collected from patients with enteric disease (cases) caused by *Campylobacter* (n=25; 41.0%), *Salmonella* (n=29; 47.5%), *Shigella* (n=4; 6.6%) and Shiga toxin-producing *E. coli* (STEC) (n=3; 4.9%) prior to treatment (**Supplementary Table S1**). After providing written informed consent, the same patients (follow-ups) submitted a second stool sample after they had recovered from the initial infection. The range of the follow-up period was 8-205 days, though the period was not known for one case. All stools were preserved in Carey-Blair transport media for submission to either the MDHHS or Michigan State University (MSU) for processing. Data about demographics, exposure history, antibiotic use, hospitalization, and symptoms were obtained from a questionnaire, or the Michigan Disease Surveillance System (MDSS) as noted in our prior study (Singh et al., 2015). Resident counties were classified as ‘rural’ or ‘urban’ based on the National Center for Health Statistics classification scheme (Ingram and Franco, 2014).

### 2.2 Sample preparation and sequencing

Metagenomic DNA was extracted, sheared, and normalized as described (Singh et al., 2015). Briefly, stools were initially homogenized and centrifuged prior to long-term storage at –80°C. The QIAamp DNA Stool Mini Kit (QIAGEN, Valencia CA) was used to extract metagenomic DNA from approximately 200 mg of fecal matter. Libraries were constructed using the TruSeq Nano library kit (Illumina, Inc., San Diego, CA, USA). DNA was fragmented prior to end-repair and 3’ adenylation. DNA adapter ligation was completed with a thermocycler program of 30°C for 10 min followed by a hold at 4°C. Subsequent DNA amplification included the following PCR program: 3 min at 95°C followed by eight cycles of 98°C for 20 s, 60°C for 15 s, and 72°C for 30 s; the final step was 5 min at 72°C. Libraries were then validated, normalized, and pooled. Shotgun metagenomic sequencing was performed in four runs on the Illumina HiSeq (Hansen et al., 2021, 2023). Samples were not selected in a specific order per batch. The Real Time Analysis (RTA) v1.18.66 (Illumina) software was used for base calling. Briefly, reads were demultiplexed at the MSU Research Technology Support Facility, converted to FASTQ files with the Bcl2fastq v2 + conversion software (Illumina). Paired-end sequences were processed using the AmrPlusPlus v2.0 pipeline (https://www.meglab.org/amrplusplus/). Trimmomatic (Bolger et al., 2014) was used to remove adapters (https://github.com/BioInfoTools/BBMap/blob/master/resources/adapters.fa) and poor-quality sequences by trimming the 5’ end of a sequence until an average Phred score > 15 was achieved with a sliding window value of 4. Sequences < 36 nucleotides were discarded. Reads matching to adapter sequences with ≤ 2 mismatches and a match score ≥ 30 were clipped to ensure complete adapter removal. Resulting reads were mapped to the human genome, GRCh38 (GRCh38_latest_genomic.fna.gz, downloaded December 2020) in RefSeq using the Burrows-Wheeler Aligner (Li and Durbin, 2009). SAMTools (Li and Durbin, 2009) and BEDTools (Quinlan and Hall, 2010) were used to remove those reads that aligned to the human genome. Non-human reads were saved as FASTQ files for use in the subsequent metabolic prediction analyses. Two paired samples were omitted from the metabolic pathway prediction pipeline due to poor alignment and annotation, though they were still included in the untargeted metabolomics analysis.

### 2.3 Metabolic prediction profiling from metagenomes

The HMP Unified Metabolic Analysis Network (HUMAnN) 3.0 program was used to profile the abundance of microbial metabolic pathways (Beghini et al., 2021). Non-host paired-end reads were merged for input into the program that used the UniRef90 database (Suzek et al., 2015) to generate gene family abundances, pathway abundances, and pathway coverage estimates for each sample. The entire gene family data file was input into the program ‘humann_infer_taxonomy’ to retrieve taxonomic information for “unclassified” genera. Resulting files were re-run through HUMAnN 3.0 to compute pathway abundances and coverage as reads-per-kilobase (RPK) values, which were normalized to relative abundances with the ‘humann_renorm_table’ function. The ‘humann_regroup_table’ function was used to display gene families with MetaCyc reaction annotations (Caspi et al., 2020), while ‘humann_rename_table’ was used to assign MetaCyc pathway names. Lastly, ‘humann_barplot’ was used to produce plots of stratified metabolic features sorted by metadata assignment or Bray-Curtis dissimilarity.

### 2.4 Ecological analyses of metagenomics data

The diversity of predicted metabolic profiles was compared between cases and follow-ups using pathway abundances from MetaCyc as input. Alpha and beta diversity metrics were calculated and plotted in R (www.R-project.org/) as was described previously (Hansen et al., 2021). To identify differentially abundant metabolic signatures in cases and follow-ups, the Meta-analysis Methods with Uniform Pipeline for Heterogeneity in Microbiome Studies (MMUPHin) was used in R (Ma et al., 2022). Batch adjustment of relative abundance data was performed based on sequencing run. Significant associations between predicted microbial pathways and health status were explored via covariate-adjusted meta-analytical differential abundance testing. Health status was included as a fixed effect, while age (years), sex (male/female), antibiotic use (yes/no) and the number of genome equivalents were all included as covariates in the model as described previously (Hansen et al., 2023) Significance values (p-values) were adjusted using the Benjamini-Hochberg method of correction for multiple hypothesis testing (q-value representing False Discovery Rate (FDR)).

MMUPHin was also used to identify intrinsic drivers of beta diversity point distributions (Ma et al., 2022). To classify potential gradients, the ‘continuous_discover()’ function was applied to the metabolic profile abundances for unsupervised continuous structure discovery using Principal Components Analysis (PCA). Upon generation of these continuous structure scores or “loadings”, plots were generated to visualize the main drivers of continuous data structure. Loadings that comprised the top principal components were compared across batches to identify “consensus” loadings for microbial features, which were overlaid onto ordination plots based on Bray-Curtis dissimilarity.

### 2.5 Metabolite extraction

Metabolite extractions were performed using 20 mg of stool from each of the 120 samples. Each sample was incubated on ice for 10 minutes after adding 350 μl of ice-cold methanol with 0.1% butylated hydroxytoluene (BHT). Five internal standard solutions for quality control and normalization included: 1) ^13^C-labeled short-chain fatty acids (SCFAs) (10 µM each of [^13^C]sodium formate, [^13^C_2_]sodium acetate, [^13^C_3_]sodium propionate, and [^13^C_4_]sodium butyrate in 50:50 (v/v) methanol/water); 2) phenylalanine-d_7_ (10 µM in 50:50 methanol/water); 3) succinic acid-d_4_ (10 uM in 50:50 methanol/water; 4) [^13^C_16_]palmitic acid (10 µM in 100% isopropanol); and 5) labeled bile acids (10 µM each of glycocholic acid-*d*_4_ and glycoursodeoxycholic acid-*d*_4_ in 50:50 methanol/water). Samples were mixed via agitation for 30 seconds and centrifuged (10,000 x g) at 4°C for 10 minutes. The supernatant was transferred to a sterile microcentrifuge tube on ice; the remaining pellet was washed with ice-cold HPLC-grade isopropanol and the sample was homogenized for 30 seconds via agitation and centrifuged again (10,000 x g) at 4°C for 10 minutes. The resulting isopropanol supernatant was combined with the initial extract to form a ‘Total Extract’ (TE) from which 100 µl were aliquoted into amber glass autosampler vials (2 mL) sealed with 9 mm screw septum caps and stored at -80°C until use.

### 2.6 Liquid chromatography mass spectrometry

Each sample was analyzed using separate reverse phase and hydrophilic interaction liquid chromotography (HILIC) methods to cover a wide range of polar and nonpolar metabolites, respectively. A Q-Exactive™ Hybrid Quadrupole-Orbitrap mass spectrometer (MS) was coupled with a Thermo Scientific™ Vanquish Ultra High-Performance Liquid Chromatography (UHPLC) system to analyze metabolites via Liquid Chromatography Mass Spectrometry (LC-MS). Blank and pooled samples were included at the beginning of each run (reverse phase and HILIC separation) and every 20 samples; three blank samples were processed between reverse phase and HILIC runs.

For reverse phase separation (polar metabolites), 10 µL of sample was injected onto a Waters Acquity Ethylene Bridged Hybrid (BEH)-C18 UPLC column (2.1x100mm) at 60°C. Compounds were separated using a gradient with a 0.4 ml/min flow rate. Initial conditions were 98% mobile phase A (water + 0.1% formic acid) and 2% mobile phase B (acetonitrile + 0.1% formic acid), hold at 2% B until 1 min, ramp to 100% B at 8 min, hold at 100% B until 10 min, return to 2% B at 10.01 min and hold at 2% B until 12 min. For HILIC separation, 10 µL of sample was injected onto a Waters BEH-Amide UPLC column (2.1x100mm) at 60°C. The gradient was run at 0.4 ml/min with initial conditions of: 100% mobile phase B (10 mM ammonium formate/10 mM ammonium hydroxide in 95:5 acetonitrile/water (v/v) and 0% mobile phase A (10 mM ammonium formate/10 mM ammonium hydroxide in water), hold until 1 min at 100% B, ramp to 40% B at 8 min, hold at 40% B until 10 min, return to 100% B at 10.01 min and hold at 100% B until 12 min.

Mass spectra were acquired for both methods using the same settings. Compounds were ionized by electrospray ionization in positive ion mode with a capillary voltage of 3.5 kV, transfer capillary temperature of 262.5°C, sheath gas at 50, auxiliary gas at 12.5, probe heater at 425°C, and S-lens RF level at 50. A data-dependent MS/MS method was used to acquire spectra with survey scan settings of 35,000 resolution, AGC target 1e6, maximum inject time 100 ms, and m/z range 100-1500. MS/MS spectra were acquired for the top 5 ions at a resolution setting of 17,500, AGC target 1e5, minimum AGC 5e3, maximum inject time 50 ms, isolation window of 1.5, fixed first mass at m/z 50, dynamic exclusion setting of 3 s and stepped normalized collision energy settings of 20, 40 and 60.

### 2.7 Data processing for mass spectrometry output

Chromatographic component separation of fecal metabolites was exported as. RAW files via Xcalibur™. Raw data were transformed to the.mzXML format with the Global Natural Product Social (GNPS) Molecular Networking conversion software (Wang et al., 2016). MS data processing was performed separately for polar and nonpolar output files using MZmine v.2.53 (Myers et al., 2017). mzXML files were imported to MZmine for centroided mass detection at MS1 and MS2 using a noise level of 1.5E05 for MS1 and 1.0E4 for MS2; these noise cutoffs were determined by visually investigating random scans pooled and blank samples. Chromatograms were constructed using the ADAP (Automated Data Analysis Pipeline) Chromatogram Builder Module (Myers et al., 2017) with a scan retention time (RT) of 1.00 - 10.00 min for MS1. A minimum group size in number of scans was set to 5, while a group intensity threshold of 1.5E5, a minimum highest intensity of 1.5E5, and a m/z tolerance (for scan-to-scan accuracy) of 0.02 m/z or 5.0 ppm were used.

Chromatograms were deconvoluted using a baseline cut-off algorithm with a minimum peak height and baseline level of 1.5E5 and a peak duration ranging from 0.00 to 0.50 min. The m/z range for MS1 and MS2 scan pairing was 0.02 Da while the RT range for MS1 and MS2 scan pairing was 0.1 min; original features were not removed during deconvolution. Isotopic peaks were grouped with an m/z tolerance of 0.02 m/z or 5.0 ppm, an absolute RT tolerance of 0.1 min, and a maximum charge of 5 with the representative isotope designated by highest peak intensity.

An aligned feature list containing data from all samples was generated using the Join Aligner method, which aligns detected peaks based on a match score determined by the mass and retention time of each peak. Settings included a m/z tolerance of 0.02 m/z or 5.0 ppm with a weight for m/z of 75, while the absolute RT tolerance was 0.1 min and the weight for RT was set to 25. The feature list was filtered by row to identify features present in at least three samples; only peaks with a MS2 scan available were kept in the feature list and peak number IDs were reset during this step. After filtering, gap filling was achieved by using the multithreaded peak finder function. The intensity tolerance was set to 10.0% with a m/z tolerance of 0.02 m/z and 5.0 ppm; the absolute RT tolerance was 0.1 min. The resulting feature list was exported for further analysis in GNPS with the Feature-Based Molecular Networking (FBMN) workflow; only rows with MS2 were included. Exported files included a feature quantification table (.CSV) and a MS/MS spectral summary file (.MGF).

### 2.8 Feature-based molecular networking of metabolites

A molecular network was created with the FBMN workflow (Nothias et al., 2020) through GNPS (Wang et al., 2016). The data were filtered by removing all MS/MS fragment ions within +/- 17 Da of the precursor m/z. MS/MS spectra were window filtered by choosing the top six fragment ions in the +/- 50 Da window. The precursor ion mass tolerance was set to 0.02 Da and the MS/MS fragment ion tolerance to 0.02 Da. A molecular network was created after filtering the edges to have a cosine score over 0.7 (nonpolar metabolites) or 0.65 (polar metabolites) with >4 matched peaks. The edges between two nodes were retained if each node appeared in both top-10 most similar node outputs. The maximum size of a molecular family was set to 100, and the lowest scoring edges were removed from molecular families until the size was below this threshold. The spectra in the network were searched against GNPS spectral libraries (Horai et al., 2010; Wang et al., 2016); the library spectra were filtered in the same manner as the input data. All matches kept between network spectra and library spectra required a score above 0.7 and at least 4 matched peaks. The DEREPLICATOR was used to annotate MS/MS spectra (Mohimani et al., 2018), while the networks were visualized internally in GNPS and externally using Cytoscape software (Shannon et al., 2003).

### 2.9 Intensity normalization and random forest

Metabolic intensities were output by FBMN for GNPS. The cluster index was used to associate peak intensities with metadata. All metabolites identified in blank samples were removed from the dataset and peak intensities were normalized via sum-scaling. The randomForest package in R (v 4.6-14) was used (Liaw and Wiener, 2002) that combines multiple decision trees into an ensemble, thereby reducing error and increasing the accuracy of assignments (Breiman, 2001). The dichotomous variable “health status”, which stratifies by cases or follow-ups, was input and samples were classified via agreement of 5,000 decision trees generated based on intensities. If a compound could not be characterized, other clusters in a related molecular network were explored for annotation. The top-30 polar and top-30 nonpolar metabolites identified by random forest were subset and used to generate heatmaps displaying the normalized intensity of clusters differentiating cases and follow-ups. Intensity values were log-transformed to appropriately scale the data for viewing.

### 2.10 Statistical analysis of metabolites

Normalized peak intensity tables for polar and nonpolar metabolites were used to assess alpha and beta diversity of the metabolomes of cases and follow-ups. Metrics such as metabolite richness, Shannon-Weiner diversity, Pielou’s evenness index, and Bray-Curtis dissimilarity were calculated and plotted in R (www.R-project.org/) as described previously (Hansen et al., 2021). Additionally, peak intensities were input to MetaboAnalyst 5.0 (Pang et al., 2021). The ‘Statistical Analysis [one factor]’ approach was used for a paired analysis of the case and follow-up samples per individual with a paired fold-change (FC) analysis and 5.0 cutoff. A volcano plot of nonparametric paired FC values was generated with the same cutoff using a FDR threshold of 0.05 with an assumption of equal variances.

## 3 Results

### 3.1 Characteristics of the study population

While all samples were included when performing untargeted metabolomics, one case/follow-up pair was removed from the metagenome analysis due to poor sequencing quality. Among the remaining 60 cases, 28 (46.7%) self-identified as male and 26 (43.3%) were between 19-64 years of age. Most cases identified as Caucasian (n=48; 80.0%), lived in urban areas (n=33; 55%), and reported abdominal pain (n=50; 84.8%) and diarrhea (n=57; 96.6%) during the initial infection. Seventeen cases (28.3%) required hospitalization and two (3.3%) reported antibiotic use within two weeks prior to the initial stool submission. The average follow-up period was 107.9 days and five (8.3%) cases reported antibiotic use within two weeks of collecting the follow-up sample.

### 3.2 Variation in the metabolic potential of the gut during and after enteric infection

**Figure 1:**
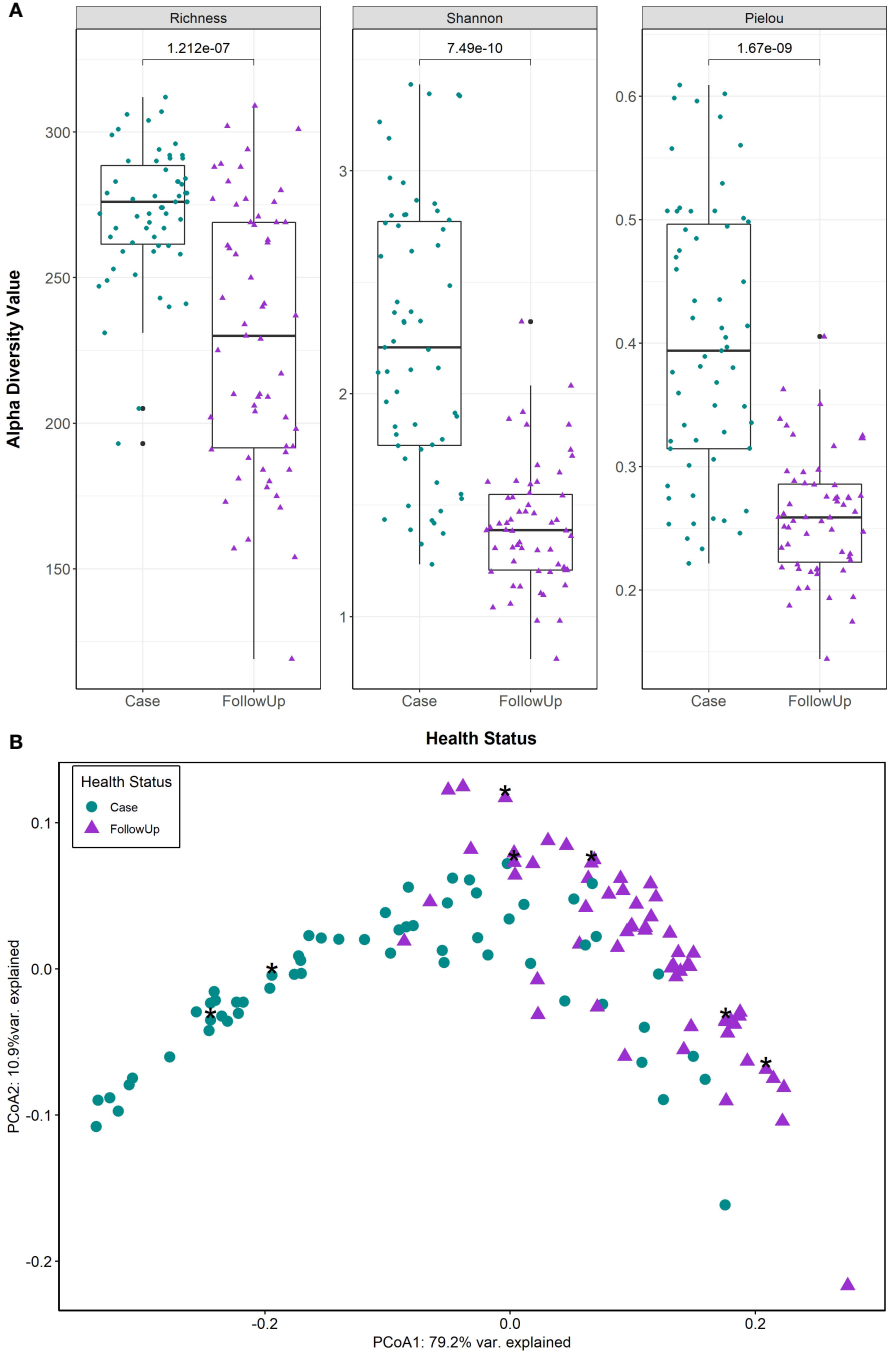
Predicted MetaCyc pathways show significant differences in metabolic potential among patients during and after enteric infection. **(A)** Alpha diversity measures (Pielou’s Evenness, Richness, and Shannon diversity) are displayed in each boxplot. Case and follow-up (FollowUp) samples are indicated by green circles and purple triangles, respectively. Data points are offset from the vertical and the median is shown as a thick black bar, while the bottom and top of each box represents the first and third quartiles. P-values are shown above the comparison bar and were calculated using the Wilcoxon signed-rank test for paired samples. **(B)** Case (green circles) and follow-up (purple triangles) samples were examined by principal coordinates analysis (PCoA) based on Bray-Curtis dissimilarity of community level pathway abundances; cases reporting antibiotics within the 2 weeks prior to sample collection are also shown as asterisks. The first and second coordinate are displayed with their respective percentage of variance explained.

HUMAnN 3.0 identified 389 MetaCyc pathways at the community level (i.e., pathways not assigned to a specific genus) in the 118 paired samples from 59 individuals; one outlier was omitted along with its paired counterpart. Cases had significantly more metabolic pathway signatures than follow-ups (S_case_=272, S_follow-up_=230 p=1.212e-07; Wilcoxon signed-rank test) as well as enhanced diversity (H’_case_=2.25, H’_follow-up_=1.41; p=7.49e-10) and evenness (J’_case_=0.402, J’_follow-up_=0.260; p=1.67e-09), (**Figure 1A**). Predicted pathway composition was also significantly different between cases and follow-ups (PERMANOVA, F=62.73; p=0.001) (**Figure 1B**) as was the level of dispersion (PERMDISP, F=20.10; p=0.001), with case samples having a greater average distance to the median (0.148) than the follow-up samples (0.0947).

The extensive overlap and arch effect in the ordination plots suggest a potential gradient (continuous structure) of metabolic pathway abundance across samples. MMUPHin was used to correct for batch effects in sequencing runs identified in our previous study (Hansen et al., 2023) to evaluate the structure. After removing unmapped reads, rhamnose biosynthesis and histidine degradation were present primarily in follow-up-like samples (**Supplementary Figure S1A**). These were opposite a superpathway for glycolysis, TCA, and glyoxylate bypass, a palmitate biosynthesis pathway, and an ornithine degradation pathway that were identified in case-like samples. The top 20 loadings (**Supplementary Table S2**) in the gradient-labeled ordination plots enables interpretation of metabolic tradeoffs driving the observed distribution of points (**Supplementary Figure S1B**). For example, a tradeoff was observed between metabolic profiles dominated by rhamnose biosynthesis and histidine degradation and those with heavy signatures of glycolysis and glyoxylate bypass, ornithine degradation, and palmitate synthesis.

### 3.3 Functional differences in metabolic pathways during and after infection

**Figure 2:**
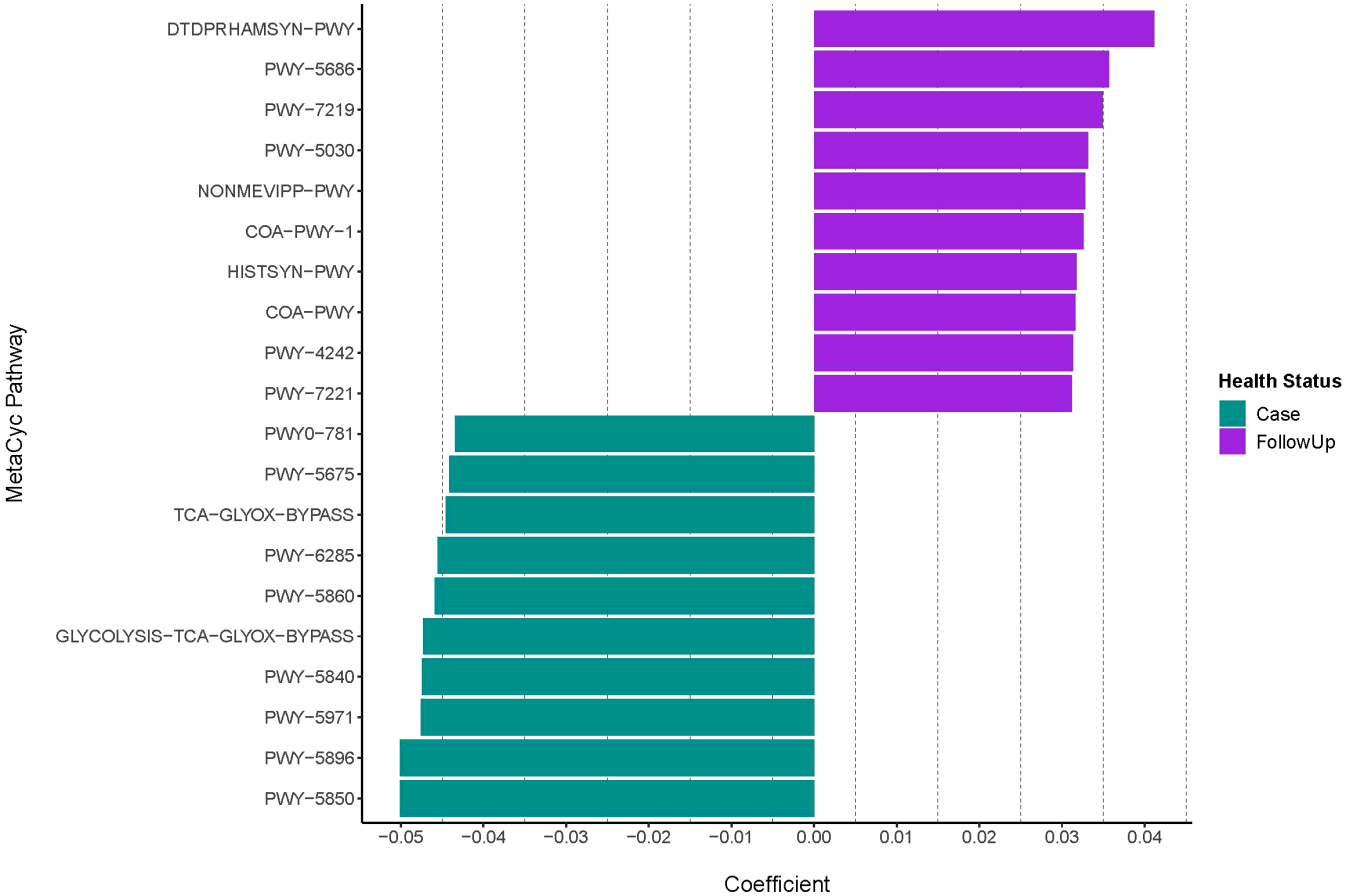
Differentially abundant MetaCyc pathways among infected and recovered samples. The case and follow-up samples had differentially abundant metabolic features that were detected with MMUPHin after removing the UNMAPPED reads. Pathway coefficients are shown on the x-axis The top-10 pathways demonstrating the strongest associations that differentiate by health status are shown. Positive coefficients indicate metabolic pathways with higher abundances in follow-ups (purple), whereas negative coefficients show pathways more represented among cases (green). Pathway names displayed on the y-axis are shortened to their BioCyc ID via MetaCyc; full pathway names, coefficients, standard error, p-values, and q-values are shown in **Table 1**.

Differential abundance analysis demonstrated that cases were primarily defined by menaquinol biosynthesis (including menquinol-10, -6, and -7), palmitate biosynthesis (coef= -0.048; q-value= 0.0061), and glycolysis, TCA, and glyoxylate bypass pathways (coeff= -0.047; q-value= 1.76e-07) (**Figure 2**; **Table 1**). By contrast, follow-up samples had a high abundance of L-rhamnose (coeff= 0.041; q-value=2.12e-10) and UMP (coef= 0.036; q-value=8.06e-08) biosynthesis pathways.

**Table 1:** Metabolic pathways identified in patients with enteric infections (Case) and post-recovery (FollowUp).

Pathway ID	MetaCyc Pathway	Class	Coefficient	Standard Error	p-value	q-value (False Discovery Rate)
**DTDPRHAMSYN-PWY**	dTDP-&beta;-L-rhamnose biosynthesis	FollowUp	0.0412	0.0062	3.46E-11	2.12E-10
**PWY-5686**	UMP biosynthesis I	FollowUp	0.0357	0.0064	2.03E-08	8.06E-08
**PWY-7219**	adenosine ribonucleotides *de novo* biosynthesis	FollowUp	0.0349	0.0038	2.81E-20	7.24E-19
**PWY-5030**	L-histidine degradation III	FollowUp	0.0332	0.0055	1.51E-09	7.48E-09
**NONMEVIPP-PWY**	methylerythritol phosphate pathway I	FollowUp	0.0328	0.0049	1.72E-11	1.09E-10
**COA-PWY-1**	superpathway of coenzyme A biosynthesis III (mammals)	FollowUp	0.0326	0.0029	1.68E-28	2.16E-26
**HISTSYN-PWY**	L-histidine biosynthesis	FollowUp	0.0318	0.0038	7.64E-17	1.13E-15
**COA-PWY**	coenzyme A biosynthesis I (prokaryotic)	FollowUp	0.0316	0.0047	1.39E-11	9.12E-11
**PWY-4242**	pantothenate and coenzyme A biosynthesis III	FollowUp	0.0314	0.0039	2.00E-15	2.08E-14
**PWY-7221**	guanosine ribonucleotides *de novo* biosynthesis	FollowUp	0.0312	0.0046	1.11E-11	7.41E-11
**PWY0-781**	aspartate superpathway	Case	-0.0434	0.0069	3.98E-10	2.10E-09
**PWY-5675**	nitrate reduction V (assimilatory)	Case	-0.0441	0.0085	2.33E-07	7.62E-07
**TCA-GLYOX-BYPASS**	superpathway of glyoxylate bypass and TCA	Case	-0.0445	0.0077	8.37E-09	3.51E-08
**PWY-6285**	superpathway of fatty acids biosynthesis (*E. coli*)	Case	-0.0455	0.0120	1.54E-04	3.46E-04
**PWY-5860**	superpathway of demethylmenaquinol-6 biosynthesis I	Case	-0.0459	0.0055	8.31E-17	1.19E-15
**GLYCOLYSIS-TCA-GLYOX-BYPASS**	superpathway of glycolysis, pyruvate dehydrogenase, TCA, and glyoxylate bypass	Case	-0.0473	0.0087	4.75E-08	1.76E-07
**PWY-5840**	superpathway of menaquinol-7 biosynthesis	Case	-0.0474	0.0079	2.25E-09	1.06E-08
**PWY-5971**	palmitate biosynthesis (type II fatty acid synthase)	Case	-0.0476	0.0162	3.31E-03	6.15E-03
**PWY-5896**	superpathway of menaquinol-10 biosynthesis	Case	-0.0501	0.0062	4.41E-16	5.00E-15
**PWY-5850**	superpathway of menaquinol-6 biosynthesis	Case	-0.0501	0.0062	4.41E-16	5.00E-15

### 3.4 Specific metabolic pathways differ between sample groups

**Figure 3:**
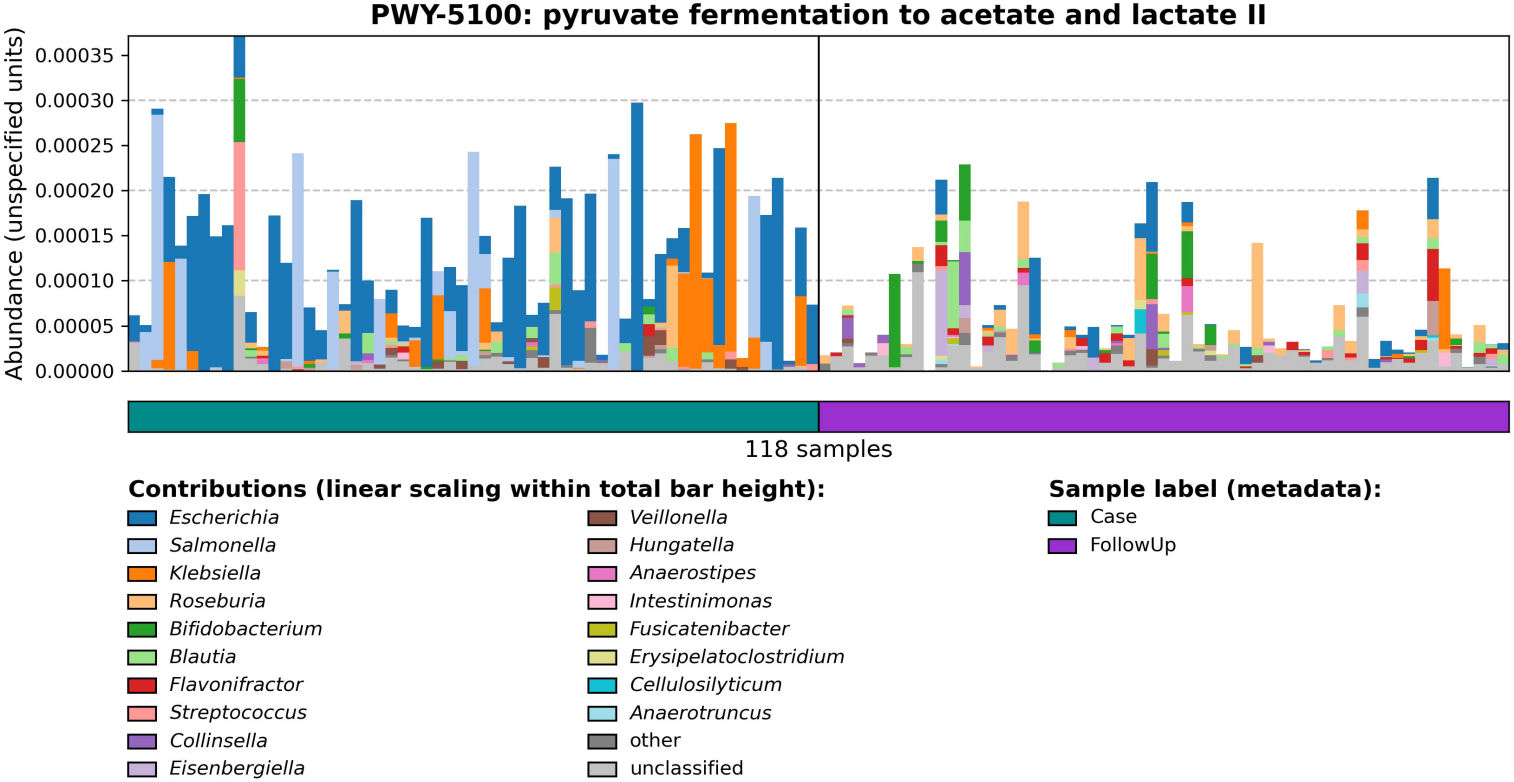
Relative abundances of PWY-5100: pyruvate fermentation to acetate and lactate II among cases and follow-ups. Barplots show the relative abundance of PWY-5100 calculated by HUMAnN 3.0. The horizontal color bar on the bottom designates case (green) vs. follow-up (purple) samples. The ‘Contributions’ section displays genera found to be associated with the pathway of interest as determined by MetaPhlan 3.0; colors in the stacked barplots show the proportion of relative abundances for PWY-5100 attributed to that specific genus.

As microbial SCFA metabolism contributes to host metabolic health, MetaCyc pathways related to compounds such as butyrate, propionate, and acetate production and degradation were explored. MetaCyc PWY-5100: pyruvate fermentation to acetate and lactate II pathway was most abundant in both sample types but was primarily associated with cases (**Figure 3**; coef= -0.0069; q-value=0.027). A pathway potentially involved in acetate synthesis, PWY-7254: TCA cycle VII (acetate-producers), was also associated with cases (coef= -0.029; q-value= 0.00069). By contrast, P163-PWY: L-lysine fermentation to acetate and butanoate, which is relevant to butyrate production, was associated with follow-ups (coeff=0.0032; q-value=0.022). Other butyrate-specific pathways included PWY-5676: acetyl-CoA fermentation to butanoate II, CENTFERM-PWY: pyruvate fermentation to butanoate, and PWY-5677: succinate fermentation to butanoate.

Although these pathways registered low relative abundance and were not differentially abundant between cases and follow-ups, the distribution of pathways among samples suggests various patterns cannot be captured by statistical analysis alone. Propionate production, for instance, was only identified in one pathway, P108-PWY: pyruvate fermentation to propanoate I, but was not associated with either sample type based on differential abundance. Nonetheless, some interesting patterns were observed for this pathway among samples and taxa. All other pathways related to butyrate, acetate, and propionate involved degradation of these compounds. In agreement with our earlier results, a pathway (PWY-5971: palmitate biosynthesis (type II fatty acid synthase)) involved in the production of palmitate, another relevant fatty acid in the human body, was prevalent primarily among cases. Relative abundances and correlation coefficients (if applicable) of SCFA-related pathways are shown in **Supplementary Table S3**.

Various metabolites that have been linked to gut dysbiosis were also explored. For example, the lipopolysaccharide (LPS) of Gram-negative bacteria is known to cause inflammation and health issues. Hence, the observation that the LPSSYN-PWY: superpathway of LPS biosynthesis was more abundant in cases (coef= -0.021; q-value=2.95e-14) is notable (**Supplementary Figure S2A**). Moreover, stratifying by causative pathogen of the acute infections led to interesting differences in taxa associated with this pathway (**Supplementary Figure S2B**). Notably, *Escherichia* was associated with the LPS biosynthesis pathway in infections caused by *Campylobacter* spp., STEC, and *Shigella* spp, while *Salmonella* spp. was most important for patients with *Salmonella* infections. Similarly, the presence of *p-*Cresol, a derivative of toluene that has carcinogenic properties, has also been linked to reduced gut health. One pathway related to *p-*Cresol production, PWY-5181: toluene degradation III (aerobic) (via p-cresol) was found in the samples. This pathway had low abundances in general (case=2.09e-05; follow-up=2.50e-06) but was affiliated with cases (coef= -0.0093; q-value=0.0055).

### 3.5 Recovered patients have greater diversity of polar and nonpolar metabolites

After filtering and normalization of mass-spectra collected via untargeted metabolomics, a total of 7,916 polar features and 13,940 nonpolar features were identified among cases and follow-ups (**Supplementary Figure S3**). Overall, the follow-up samples had significantly greater richness of polar metabolites than the case samples (S_case_=875, S_follow_=1024 p=2.28e-07; Wilcoxon signed-rank test), though no difference in Shannon diversity was observed (H’_case_=5.00, H’_follow_=5.07; p=0.8971; **Supplementary Figure S4A**). The cases, however, had greater evenness of the polar metabolites (J’_case_=0.739, J’_follow_=0.731; p=0.008211, respectively), which clustered distinctly in the PCoA plot by sample type (PERMANOVA F-value=26.27; p-value=0.000999) despite the greater dispersion among cases (PERMDISP p-value=0.026; **Supplementary Figure S4B**).

In contrast, the nonpolar metabolites were significantly more diverse in the follow-up samples across all three measures (S_case_=1790, S_follow_=2832, p=1.53e-11; H’_case_=4.89, H’_follow_=5.96, p=1.48e-10; J’_case_=0.656, J’_follow_=0.750, p=6.49e-09; **Supplementary Figure S5A**). Similarly, the cases and follow-ups clustered separately based on Bray-Curtis dissimilarity of nonpolar metabolite composition (PERMANOVA F-value=19.607; p-value=0.000999); PERMDISP indicated a significant difference in the dispersion of points (F-value=14.903; p-value=0.001), particularly for the cases (**Supplementary Figure S5B**).

When the samples were stratified by the two predominant pathogens, *Campylobacter* and *Salmonella*, no significant differences were observed in diversity of polar metabolites. Cases infected with *Campylobacter*, however, had greater nonpolar metabolite diversity during infection when compared to those cases with *Salmonella* infections (S*_Campylobacter_* =2021, S*_Salmonella_* =1627, p=0.032; H’*_Campylobacter_* =5.18, H’*_Salmonella_* =4.64, p=0.0085; J’*_Campylobacter_* =0.683, J’*_Salmonella_* =0.631, p=0.024; **Supplementary Figure S6A**). Nonetheless, distinct clustering by causative pathogen was not observed when the polar metabolite composition was examined (PERMANOVA F-value=1.260; p=0.1209) despite the significant dispersion of points (PERMDISP F-value=4.549, p=0.013; **Supplementary Figure S6B**). Similar results were observed for the nonpolar metabolite composition (PERMANOVA F-value=1.2301, p=0.1189; PERMDISP F-value=3.263, p=0.019; **Supplementary Figure S6C**).

### 3.6 Random forest analysis identified specific polar metabolites that differentiate the infected and recovered samples

**Figure 4:**
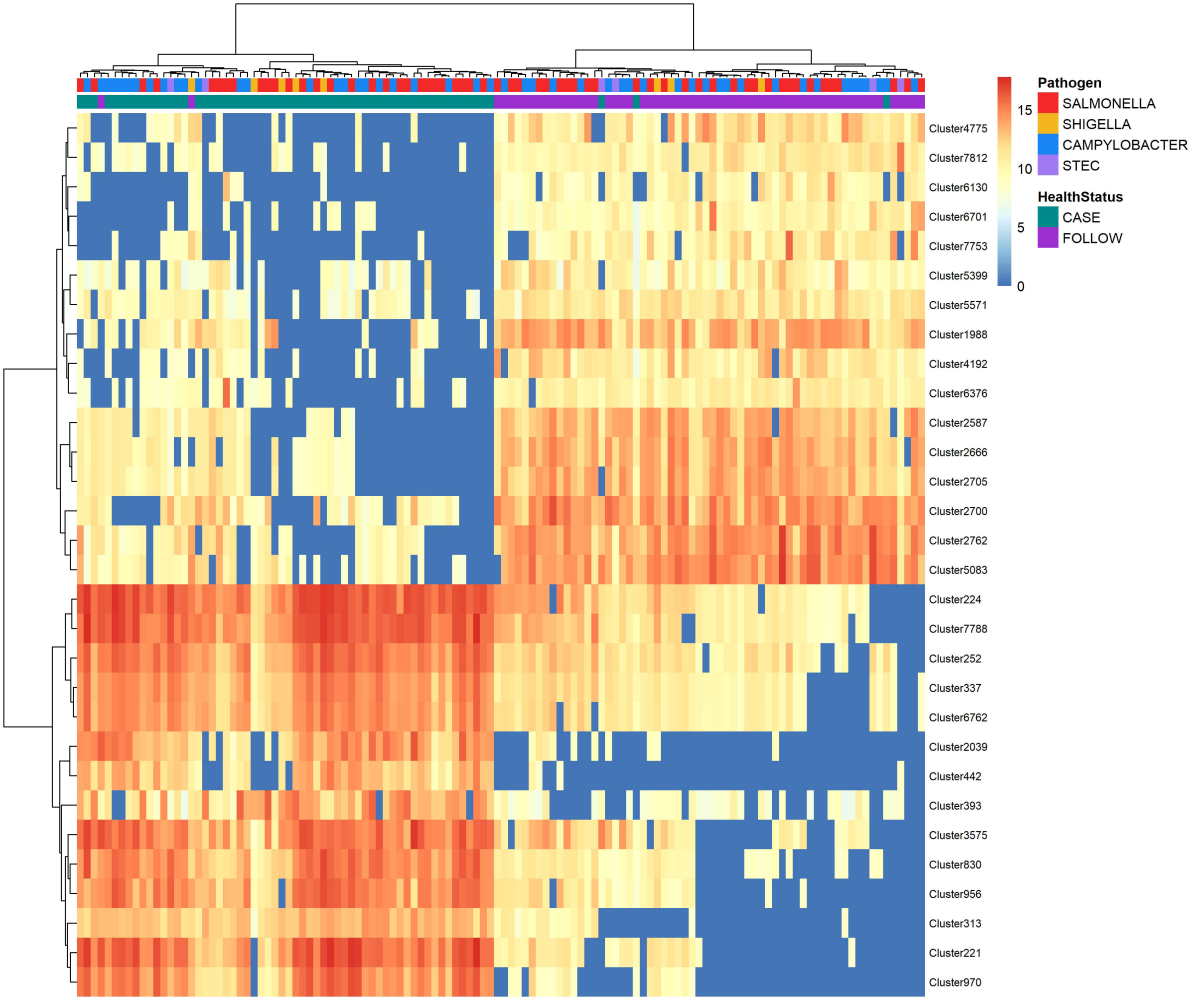
Heatmap displaying abundance of the top-30 polar metabolites identified by random forest analysis to distinguish between cases and follow-ups. The abundance of the top-30 distinguishing polar metabolites among cases and follow-ups clustered by health status. Abundances were amplified by 10^9^ and log-transformed to display in the heatmap. The color of each cell represents the abundance; metabolites of high abundance are shown in red while metabolites of low or zero abundance are shown in blue. Each column represents one sample and samples were hierarchically clustered using the Ward D2 algorithm based on the Euclidean distance of samples’ polar metabolite composition. Two color bars at the top of the heatmap represent cases (green) and follow-ups (purple), while another designates the pathogen associated with case infections (*Salmonella* – red; *Campylobacter* – blue; *Shigella* – yellow; and STEC – light purple). Rows represent metabolic features or “clusters” which are named on the right y-axis. A dendrogram was generated for these clusters based on their distribution across samples and is shown on the left y-axis.

Random forest of normalized peak intensities was used to identify polar features that could distinguish between the case and follow-up samples. Among the most important (top-30) polar metabolites predicted by random forest, the samples clustered together based on the patient’s health status (case vs. follow-up) with minimal overlap (**Figure 4**). Despite this, no clustering was observed among the cases when stratified by the infecting pathogen or among the follow-up samples when stratified by the pathogen linked to the original infection. Nonetheless, a few case clusters containing both *Salmonella* and *Campylobacter* samples were apparent, while all but one *Shigella* sample grouped within two case clusters with *Salmonella* samples.

**Figure 5:**
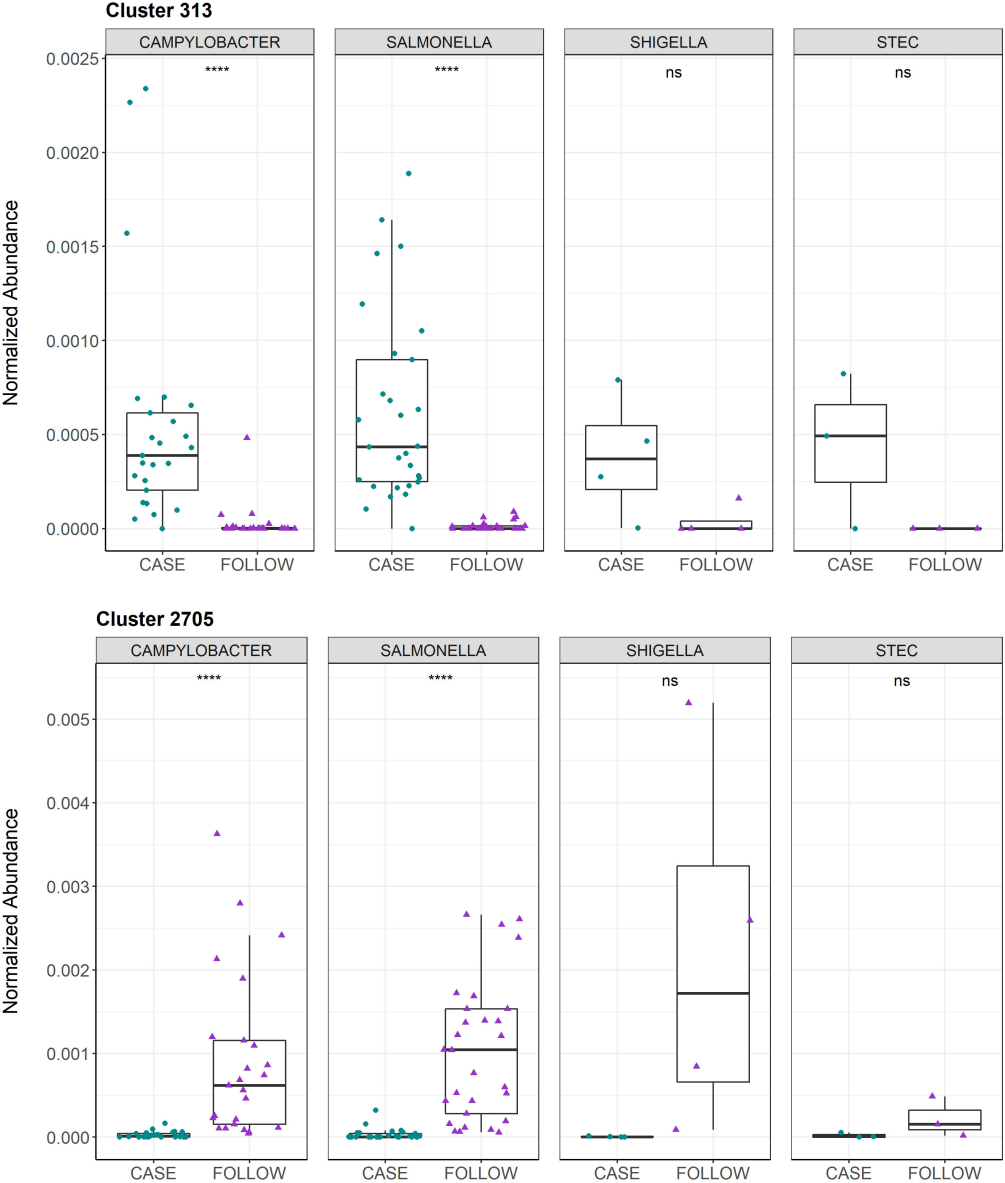
Normalized abundances for Cluster 313 and Cluster 2705 among cases and follow-ups separated by infecting pathogen. Normalized abundances of Cluster 313 (top) and Cluster 2705 (bottom) are displayed. The box-plots are faceted by infecting pathogen and stratified by health status, with samples represented by circles (cases, green) or triangles (follow-ups, purple). Data points are offset from the vertical to allow for clear interpretation of all samples. Within each box, the median is displayed as a thick black bar; the first and third quartiles are shown by the bottom and the top of each box, respectively. Significance levels are displayed on the plot and were calculated using the Wilcoxon signed-rank test for paired case and follow-up samples; ns: p>0.05, ****: p ≤ 0.0001.

The top-30 polar clusters identified in this analysis are listed in **Supplementary Table S4** with library IDs, though 24 of the 31 clusters could not be classified. The out-of-bag (OOB) estimate of error rate for our random forest classification was 5.74% for polar metabolites, suggesting high accuracy in classifying the samples based on metabolite composition. Polar Cluster 313 was deemed most important in distinguishing cases from follow-ups and registered a mean decrease in accuracy (MDA) score of 13.86. This compound was identified as {[2-hexadecanamido-3-hydroxyoctadec-4-en-1-yl]oxy}[2-(trimethylazaniumyl)ethoxy]phosphinic acid (**Supplementary Figure S7**). Although it was elevated in the case samples relative to the follow-ups for both *Campylobacter* and *Salmonella* infections (**Figure 5**), this compound was not more abundant in *Campylobacter* case samples when compared to the *Salmonella* case samples (Wilcoxon rank-sum test, p=0.85). Moreover, no association was observed between the presence of Cluster 313 and epidemiological data, such as hospitalization or reports of bloody stool (data not shown). The next most important compound in distinguishing disease status was polar Cluster 2705 (MDA=13.27), an uncharacterized compound that was elevated among the follow-up samples (**Supplementary Figure 5B**). Unlike Cluster 313, the follow-up samples had a higher abundance of Cluster 2705 relative to the cases but no difference in abundance was observed between the samples from the recovered patients with *Campylobacter* versus *Salmonella* infections (Wilcoxon rank-sum test, p=0.19).

### 3.7 Specific nonpolar metabolites also distinguish infected and recovered samples

**Figure 6:**
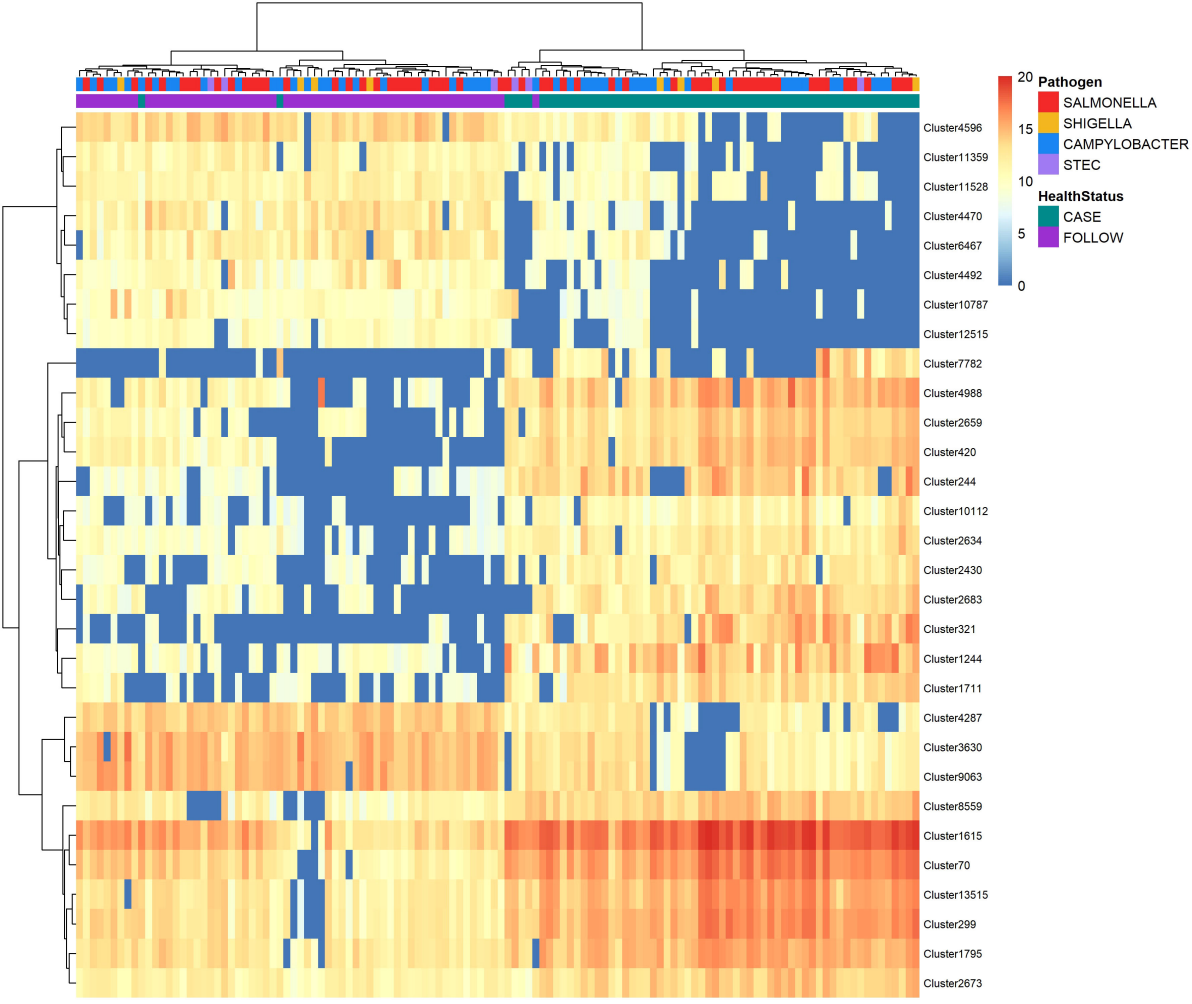
Heatmap displaying abundance of the top-30 nonpolar metabolites identified by random forest analysis to differentiate between cases and follow-ups. The abundance of the top-30 distinguishing nonpolar metabolites among cases and follow-ups clustered by health status. Abundances were amplified by 10^9^ and log-transformed to display in the heatmap. Colors represent the abundance; metabolites of high abundance are red while metabolites of low or zero abundance are blue. Each column represents one sample and samples were hierarchically clustered using the Ward D2 algorithm based on the Euclidean distance of samples’ polar metabolite composition. Two color bars at the top of the heatmap represent cases (green) and follow-ups (purple) and type of case infections (*Salmonella* – red; *Campylobacter* – blue; *Shigella* – yellow; and STEC – light purple). The right y-axis represents metabolic features or “clusters” and the left x-axis dendrogram was generated for these clusters based on their distribution across samples.

A set of the top-30 nonpolar metabolites that could distinguish between the case and follow-up samples was also identified using random forest analysis (**Figure 6**). Like the polar metabolites, the samples clustered by health status (case vs. follow-up) with minimal overlap. Similarly, the *Shigella* cases grouped together within two clusters with the exception of one sample, while the *Salmonella* and *Campylobacter* cases were intermingled among multiple clusters.

Surprisingly, only two of the top-30 nonpolar metabolites were known compounds (**Supplementary Table S5**). The OOB estimate of error rate for the nonpolar metabolites was 4.92%, suggesting that the classification of our samples based on the nonpolar metabolite composition is also highly accurate. The top-3 clusters, Cluster 2659 (MDA=12.34), Cluster 321 (MDA=11.70) and Cluster 299 (MDA=11.58), were unknown but more abundant in the case samples and could be used to differentiate the case and follow-up samples in the random forest model. Moreover, all three clusters were more abundant in the *Campylobacter* and *Salmonella* case samples relative to the paired follow-up samples (**Supplementary Figure S8**). A trend of increased abundance was observed between the STEC and *Shigella* case samples, though the number of samples evaluated was small and likely limited our ability to detect differences.

**Figure 7:**
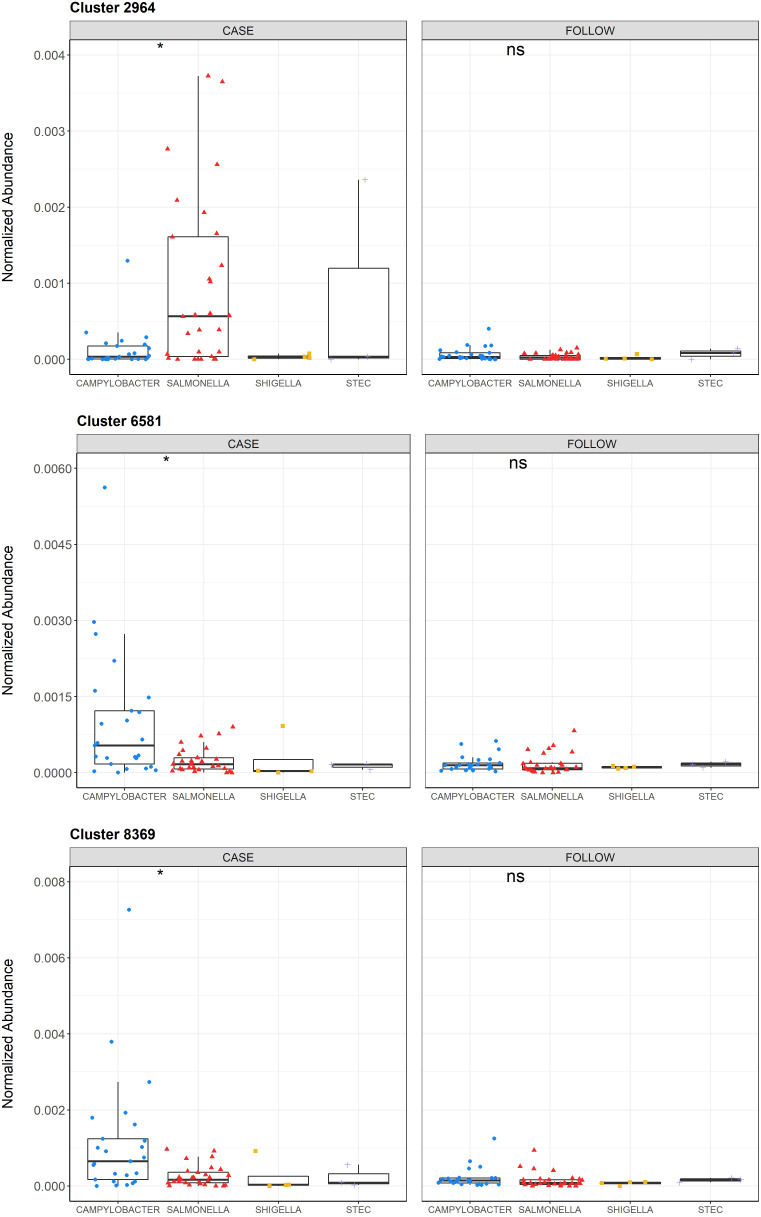
Normalized abundances of Clusters 2964, 6581, and 8369 across infecting pathogens among cases and follow-ups. Normalized abundances of Cluster 2964, 6581, and 8369 are displayed for the samples from cases (left) and follow-ups (right) and stratified by the infecting pathogen within each boxplot. The abundances of each cluster among the cases with *Campylobacter* (blue circles), *Salmonella* (red triangles), *Shigella* (yellow squares), and STEC (purple crosses) are shown. Data points are offset from the vertical and the median is represented within each box as a line. The first and third quartiles are indicated by the bottom and the top of each box, respectively. P-values were calculated using the Wilcoxon signed-rank test for paired samples and P-value signifiers are: not significant (ns): p>0.05, *: p ≤ 0.05. Statistical comparisons were only conducted for Campylobacter and Salmonella due to small sample sizes in the other two groups.

Because of these differences as well as the finding that nonpolar metabolite diversity differed by pathogen (**Supplementary Figure S6A**), we performed a separate random forest analysis to identify key metabolites that could differentiate samples from patients infected with specific pathogens. The OOB estimation of error rate was much higher for this model (41.8%), which may be partially explained by the difference in sample sizes across pathogens. Nonetheless, various metabolites could distinguish between the two predominant pathogens, *Campylobacter* and *Salmonella* (**Figure 7**). For example, nonpolar Cluster 2964 had the highest mean decrease in accuracy (6.05) and was elevated among cases infected with *Salmonella*, while nonpolar Clusters 6581 and 8369 (MDA=6.02 and MDA=5.69, respectively) were more abundant in cases infected with *Campylobacter*. Importantly, these metabolites could also differentiate between infected vs. recovered samples for the respective pathogens (**Supplementary Figure S9**). A slight increase in Cluster 8369 abundance among cases relative to follow-ups was also observed.

### 3.8 Feature-based molecular networking promotes exploration of polar and nonpolar metabolites of interest

Investigation of paired statistical analyses using MetaboAnalyst v5.0 further characterized associations between different polar and nonpolar features in the case versus recovered samples. A fold-change (FC) analysis detected metabolites present in one group or the other.

*3.8.1. Investigation of polar metabolites.* Among the polar metabolites detected, 497 were increased in the follow-ups relative to cases (i.e., a positive log_2_FC value) and 242 were increased among the cases versus follow-ups, yielding a negative log_2_FC value (**Supplementary Figure S10**). The top-25 polar metabolites with positive and negative values are listed in **Supplementary Table S6**.

**Figure 8:**
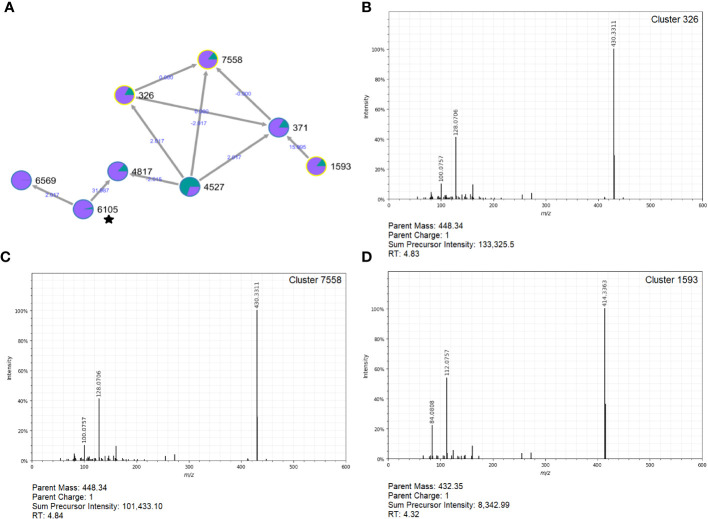
Molecular network and MS^2^ spectra for three clusters related to tomatidine that are prevalent in follow-ups. **(A)** The molecular network constructed in GNPS (top, left) shows the interrelatedness of multiple metabolite clusters. Nodes are labeled with their cluster index (black) and edges are labeled with the associated mass difference between two connected nodes (blue); directionality of the mass difference is indicated by the direction of the arrow. Pie-charts on each node indicate the proportion of that node that was found in cases (green) and follow-ups (purple). The MS^2^ spectra for Clusters 326 **(B)**, 7558 **(C)**, and 1593 **(D)** are shown; each was found to be important indicators of follow-ups. Notably, this molecular network contained metabolites related to tomatidine (Cluster 6105) designated with a star.

Notably, three clusters in the top-10 list of metabolites linked to follow-ups were part of a molecular network with tomatidine (**Figure 8A**). These included Cluster 326 (log_2_FC=8.95; p=2.42e-07; **Figure 8B**), Cluster 7558 (log_2_FC=8.32; p=6.33e-07; **Figure 8C**), and Cluster 1593 (log_2_FC=6.93; p-value=3.76e-07; **Figure 8D**). Other polar clusters increased in follow-ups included Cluster 2113 (log_2_FC=7.72; p=2.27e-08), a compound related to desmethylenylnocardamine and Nonaethylene glycol, and Cluster 2666 (log_2_FC=6.51; p=3.14e-09) related to 1-(1Z-Hexadecenyl)-sn-glycero-3-phosphocholine.

Of the polar clusters affiliated with cases, the strongest signals were from polar Cluster 970 (log2FC= -8.72; p=6.22e-09) and Cluster 221 (log2FC= -8.65; p=5.56e-09), which were in the same molecular network. This network contained multiple annotated compounds, but Clusters 970 and 221 were both directly connected to Cluster 318, which was a spectral match for 1-(1Z-Octadecenyl)-sn-glycero-3-phosphocholine (**Supplementary Figure S11**). Cluster 221 also had connections to four other nodes annotated as variations of glycerophosphocholine compounds including Cluster 227 (Lyso-PAF C-18), Cluster 1337 (1-Heptadecanoyl-sn-glycero-3-phosphocholine), Cluster 6245 (1-Hexadecyl-sn-glycero-3-phosphocholine), and Cluster 259 (sn-glycero-3-phosphocholine). Notably, each of these clusters were also in the top-30 most important polar features identified in the random forest classification for distinguishing between cases and follow-ups (**Supplementary Table S4**).

*3.8.2. Investigation of nonpolar metabolites.* Among the nonpolar metabolites detected, 1,698 increased in follow-up samples while 187 were solely affiliated with cases (**Supplementary Figure S12**). The strongest association for the case samples was the nonpolar singleton Cluster 321 (log_2_FC= -8.46; p=1.38e-08) (**Supplementary Figure S13**), which was similar to our findings generated by random forest. The lack of similarity to other metabolites in our dataset coupled with its uncharacterized nature suggests Cluster 321 may be an important, novel metabolite connected to enteric infection.

**Figure 9:**
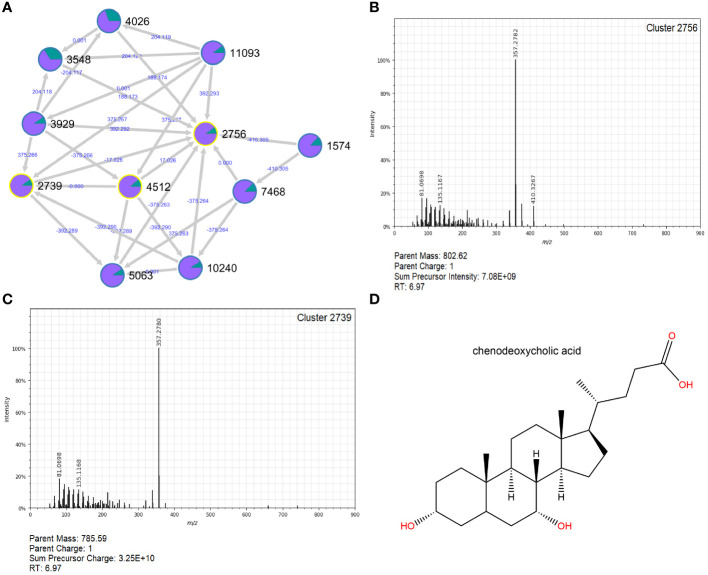
Molecular network and MS^2^ spectra for Cluster 2756 and related Cluster 2739, which were greatly increased in follow-ups. A molecular network constructed in GNPS **(A)** shows the interrelatedness of multiple metabolite clusters. Nodes are labeled with their cluster index (black) and edges are labeled with the associated mass difference between two connected nodes (blue); directionality of the mass difference is indicated by the direction of the arrow. Pie-charts on each node indicate the proportion of that node that was found in cases (green) and follow-ups (purple). The MS^2^ spectra for Cluster 2756 **(B)** and a closely related cluster, 2739 **(C)**, are shown. Clusters 2739 and 4512 (spectra not shown) were successfully annotated in GNPS as chenodeoxycholic acid; the structure for this compound was generated in ChemDraw 20.1 **(D)**.

Among the nonpolar clusters increased in follow-ups, Clusters 2756 (log_2_FC=7.03; p=3.23e-07), 4470 (log_2_FC=6.91; p=1.38e-09) and 5193 (log_2_FC=6.59; p=7.41e-08) were the most notable (**Supplementary Table S7**). Cluster 2756 was a part of an extensive molecular network comprising ten different connections. Two of these connections, Clusters 2739 and 4512, were annotated as chenodeoxycholic acid, suggesting that Cluster 2756 may be involved in the metabolism of this bile acid (**Figure 9**).

Molecular networks for the nonpolar metabolites suggested to be associated with specific enteric infections were also explored. Cluster 2964, which was more specific for *Salmonella* infections, is part of a small molecular network containing three similarly related, but uncharacterized, compounds. Comparatively, Clusters 6581 and 8369 were elevated in *Campylobacter* case samples and are part of an extensive molecular network containing 81 total compounds (**Supplementary Figure S14**). Both Clusters 6581 and 8369 were identified as glutamylphenylalanine (isomer of 1503).

## 4 Discussion

The metabolic health of the human gut is undoubtedly linked to the microbiome. Environmental flux related to disease state, diet, antibiotic use, and other factors can also greatly influence the composition of microbially-mediated metabolic pathways in the gut (Rowland et al., 2018). Taxonomically diverse gut communities, which typically represent a healthy, homeostatic gut environment (Kriss et al., 2018), have greater metabolic functionality than communities with fewer members. In our analysis, the functional prediction of microbial metabolic pathways using metagenomic data showed that patients with acute enteric infections had greater metabolic capacity during infection than post-recovery. Because we previously observed lower taxonomic diversity in the cases relative to the follow-ups (Hansen et al., 2023), this finding differs from our hypothesis that the overall metabolic capacity (i.e., number of pathways) would be similar or lower during an infection. Nonetheless, the opposite was true in our corresponding LC/MS analysis of metabolites, which showed increased metabolic diversity among the follow-up samples. The overlap between the predicted microbial metabolic pathways and the identified metabolites was relatively scant, which is likely due to the different targets for the two methods.

Perturbations in the microbiota during infection are likely to influence the abundance of microbial metabolic pathway genes. Our prior analysis showed that individuals with enteric infections had an increased abundance of antibiotic resistance genes harbored by members of *Enterobacteriaceae* such as *Escherichia* and *Klebsiella* (Hansen et al., 2023). Moreover, these genera were increased in abundance during infection regardless of the pathogen that was linked to the initial infection. Expansion of *Enterobacteriaceae* during enteric infections has previously been linked to the host-mediated inflammatory response (Lupp et al., 2007). Increased inflammation may be due, in part, to *Enterobacteriaceae*-mediated production of LPS (Caradonna et al., 2000). Support for this hypothesis comes from the greater LPS production pathway abundance observed in infected case samples. Nonetheless, the actual presence of LPS was not confirmed and would require use of additional methods such as enzyme-linked immunosorbent assays (Martinez-Sernandez et al., 2016) or direct LPS purification and quantification as described (d'Hennezel et al., 2017). Other studies have shown that nitrate, which is generated by the host during inflammation, confers a growth advantage to members of *Enterobacteriaceae* that can degrade non-fermentable substrates unlike many commensal anaerobes (Winter et al., 2013). Interestingly, we observed increased abundance of pathways with signatures of nitrogen metabolism related to nitrate reduction among the cases (**Supplementary Figure S15**) as well as pathways for amino acid regulation and biosynthesis. Multiple arginine and ornithine pathways were also detected, and these compounds are precursors for nitric oxide (NO) and polyamines (Cynober, 1994) Indeed, NO favored the overgrowth of *Enterobacteriaceae* and modified amino acid composition and concentration, and led to decreased abundance of beneficial SCFA-producing bacteria such as *F. prausnitzii* (Leclerc et al., 2021). The observed overrepresentation of these metabolic pathways in our dataset highlights the impact of increased *Enterobacteriaceae* abundance and its importance during acute infections.

Among infected cases, other notable findings included several menaquinol synthesis pathways. Menaquinols are the reduced form of menaquinones (vitamin K_2_), which facilitate electron transfer and oxidative phosphorylation in bacterial cell membranes (Collins and Jones, 1981). Increased menaquinol synthesis during enteric infection is plausible, as these compounds reduce harmful inflammatory effects and protect bacterial cell membranes from oxidation. Because enteric infection often results in inflammation, which increases luminal oxygen (Zeng et al., 2017), the elevated number of menaquinol synthesis pathways that enhance survival in these conditions is logical.

Cases also had an increased number of glycolysis, pyruvate dehydrogenase, tricarboxylic acid cycle, and glyoxylate bypass superpathways relative to the follow-ups. Accordingly, proteins for these metabolic pathways were enhanced in individuals treated with antibiotics in a prior study (Perez-Cobas et al., 2013), suggesting that microbial communities respond to fluctuating nutrient supply and stress by overcompensating in carbohydrate metabolism. The increased prevalence of glyoxylate bypass is particularly relevant, as this pathway enables microbes to consume a variety of substrates for central carbon metabolism including fatty acids, alcohols, esters, alkenes, and other compounds (Cronan and Laporte, 2005). Since antibiotic treatment causes comparable disturbances in gut microbial communities (Zeng et al., 2017), a similar effect is likely to occur during enteric infection.

The application of untargeted metabolomics also revealed distinct metabolome profiles among the case and follow-up samples. Of note, a series of glycerophosphocholines, which were assigned to at least six annotated molecules (Clusters 806, 318, 227, 259, 1337, and 6245) were variably present with 5/6 occurring more frequently in cases. Our pathway prediction pipeline identified a phosphatidyl choline acyl editing pathway, PWY-6803, to be more abundant in cases along with an overall enhanced capacity for lipid and fatty acid metabolism (**Supplementary Figure S16**). Indeed, glycerophosphocholines are required in the synthesis of phosphatidylcholine, an abundant phospholipid that plays an important role in lipid metabolism (van der Veen et al., 2017). While choline is an essential nutrient that assists with healthy brain function, cell signaling, lipid movement, and metabolism (Goh et al., 2021), it can also be metabolized by anaerobic bacteria in the gut, resulting in the generation of trimethylamine (TMA) (Wright, 2019). TMA can be metabolized by the host to form trimethylamine N-oxide (TMAO), a compound that has been linked to disease. Although TMA and TMAO were not directly detected in the samples, two trimethyl-ammonium-related products were identified in the polar metabolite analysis. One of these products, 3-hydroxy-2-(tetracosa-11.13.15)octadecyl (2-(trimethylammonio)ethyl) phosphate (Cluster 806), was elevated in cases and the other, [2-hexadecanamido-3-hydroxyoctadec-4-en-1-yl]oxy[2-(trimethylazaniumyl) ethoxy]phosphinic acid (Cluster 313), was observed to differentiate the cases from follow-ups. Since Cluster 313 was detected in most case samples (n=58; 95.1%), characterization of this compound is needed to determine its role in TMA(O) metabolism during acute infection.

Among follow-ups, Clusters 326, 7558, and 5193 were found in 66% of the samples with elevated average relative intensity compared to the cases (0.048% vs. 0.0017%, respectively). These compounds are related to a distant cluster that was annotated as tomatidine, which is the aglycon form of tomatine, a steroidal glycoalkaloid produced by members of the Solanaceae plant family (Boulanger et al., 2015). This compound is known for its benefits to human health and was shown to have anti-inflammatory and antimicrobial effects. Moreover, tomatidine is structurally similar to taurochenodeoxycholic acid (TCDCA), a conjugated bile acid with antimicrobial activity in the gut (Guthrie et al., 2019), hypothesized to acidify bacterial cells. Although the overall impact of tomatidine in the gut following an enteric infection is not known, its presence in recovered patients is intriguing and may indicate a role in a healthy, homeostatic gut environment.

Detection of the uncharacterized Cluster 2756 in follow-up samples is also intriguing, as it has connections to Clusters 2739 and 4512, identified as chenodeoxycholic acid (CDCA), a naturally occurring primary bile acid that assists with cholesterol breakdown (Iser and Sali, 1981). Gut microbes are known to facilitate important biotransformations of bile acids (Ridlon et al., 2006). Patients with irritable bowel syndrome (IBS), for instance, had increased abundance of *E. coli* and significantly more primary bile acids than their healthy counterparts, emphasizing the importance of microbial conversion of primary to secondary bile acids on gut health (Duboc et al., 2012). While lithocholic acid was slightly more common in the follow-up samples (data not shown), our findings do not clearly illustrate a predominance of secondary bile acids in the recovered samples. This discrepancy may be due to the variable timing for collecting the follow-up samples across patients, which ranged from 1 week to 29 weeks after acute infection.

It is important to note that studies utilizing untargeted metabolomics via LC/MS are limited by the uncharacterized nature of many polar and nonpolar compounds detected (Johnson and Gonzalez, 2012). While the lack of annotation limits our ability to make biologically sound conclusions, observing compositional differences among infected and recovered metabolomes is still meaningful. Each compound isolated in this study registered unique MS^2^ spectra and may lead to future characterization as metabolite databases improve. Additionally, defining the relationship between known metabolites and the unknown compounds via FBMN analysis allows us to generate hypotheses about their contribution to metabolic functions, as they may serve as precursors or intermediates in known pathways. Although we may not know the identities of metabolites that are changing in abundance, we can confidently assert that infection does play an important role in dictating the metabolic capacity of the gut.

While functional prediction of microbial metagenomes allows us to visualize metabolic capacities among gut microbes, untargeted metabolomics captures the entire metabolic chemistry of the gut environment, microbially related or not. Because untargeted metabolomics considers human-, drug- and food-derived compounds in addition to microbial-derived molecules, this method will inherently provide different results from a microbial metagenome analysis. Yet, the comparison of functional prediction with known metabolite signatures enables us to further characterize the relative importance of these microbial functions in the gut metabolome. While direct comparison of our predictive and quantitative methods is challenging, we performed a preliminary analysis to identify microbially-produced metabolites in the untargeted metabolomics dataset by comparing characterized metabolic hits in the Microbial Metabolites Database (MiMeDB) (Wishart et al., 2023). In total, just 59 characterized metabolites (35 polar, 24 nonpolar) were identified to be of microbial origin (**Supplementary Table S8**). Potential KEGG pathways related to these microbially-produced metabolites include amino acid synthesis and degradation pathways and nucleotide metabolism, demonstrating concordance with our predicted pathway pipeline. Regardless, more rigorous methods for identifying microbially produced polar and nonpolar metabolites from untargeted metabolomics are needed. Future work could include use of a taxonomically-informed mass spectrometry (MS) search tool such as microbeMASST (Zuffa et al., 2024), in conjunction with GNPS analysis to more confidently assert whether metabolites (characterized or not) are microbial in origin. In addition to highlighting the importance of comparing multiple characterization methods, these observed differences also indicate that enhanced diversity of host-derived metabolites is important for human health.

Further investigation of these data is encouraged, and an important future direction will be performing targeted metabolomics to confirm metabolites of interest identified through the study. Another analysis that should be pursued is direct integration of microbiome data with the metabolomics data described. Prior studies have demonstrated that individuals with differing microbiome compositions shared a majority of metabolic pathways identified (The Human Microbiome Project Consortium, 2012) and have described associations between various microbial taxa, predicted pathways, and fecal metabolite frequency (Visconti et al., 2019). Indeed, integrating these two ‘omics techniques (metagenomics and metabolomics) can provide a comprehensive understanding of the human gut environment. Clarifying the links between microbial composition, metabolic pathway prediction, and metabolite abundance provides a more comprehensive understanding of the changes taking place in the gut environment related to enteric infection and dysbiosis.

## Data availability statement

The sequencing datasets analyzed for this study can be found in the NCBI repository under BioProjects PRJNA862908 and PRJNA660443 (BioSamples SAMN29999523 to SAMN29999673 and SAMN15958881 to SAMN15958950, respectively). Mass spectrometry data were deposited in two separate public repositories (nonpolar: MassIVE MSV000088926; polar: MassIVE MSV000088927). The molecular networking job can be publicly accessed at https://gnps.ucsd.edu/ProteoSAFe/status.jsp?task=c20a1f9cacf1493188bdb3917dcfae69 for the nonpolar metabolites or for the polar metabolites at: https://gnps.ucsd.edu/ProteoSAFe/status.jsp?task=56654221cfd74b3db13d3ebb2d701742. All bioinformatics pipeline and analysis scripts can be found on GitHub at https://github.com/ZoeHansen/PAPER_Frontiers_Cellular_and_Molecular_Microbiology_2024.

## Ethics statement

The studies involving humans were approved by the Institutional Review Boards at MSU (IRB #10-736SM), the MDHHS (842-PHALAB), and the four participating hospital laboratories. The studies were conducted in accordance with the local legislation and institutional requirements. Written informed consent for participation in this study was provided by the participants’ legal guardians/next of kin when applicable.

## Author contributions

ZH: Conceptualization, Data curation, Formal analysis, Investigation, Methodology, Validation, Visualization, Writing – original draft, Writing – review & editing. AS: Conceptualization, Investigation, Methodology, Resources, Supervision, Validation, Writing – original draft, Writing – review & editing. DG: Conceptualization, Methodology, Visualization, Writing – review & editing. JR: Conceptualization, Funding acquisition, Investigation, Project administration, Supervision, Writing – review & editing. RQ: Conceptualization, Formal analysis, Investigation, Methodology, Resources, Supervision, Writing – review & editing. KV: Conceptualization, Formal analysis, Methodology, Visualization, Writing – review & editing. SM: Conceptualization, Data curation, Funding acquisition, Investigation, Project administration, Resources, Supervision, Validation, Writing – review & editing.
