# Supplementary material for: Shifts in the functional capacity and metabolite composition of the gut microbiome during recovery from enteric infection
Source: Front Cell Infect Microbiol. 2024 May 8;14:1359576. doi: 10.3389/fcimb.2024.1359576 (PMC11109446; doi:10.3389/fcimb.2024.1359576)
Supplement: Supplementary file 1 [file DataSheet_1.pdf]

Supplementary Material

Shifts in the functional capacity and metabolite composition of the gut microbiome during recovery from enteric infection

Zoe A. Hansen[1*†], Anthony L. Schilmiller[2], Douglas V. Guzior[1,3], James T. Rudrik[4], Robert A. Quinn[3], Karla A. Vasco[1‡], and Shannon D. Manning[1*]

[1]Department of Microbiology and Molecular Genetics, Michigan State University, E. Lansing, MI 48109, USA
[2]Research Technology Support Facility, Mass Spectrometry and Metabolomics Core, Michigan State University, E. Lansing, MI 48109, USA
[3]Department of Biochemistry and Molecular Biology, Michigan State University, E. Lansing, MI 48109, USA
[4]Michigan Department of Health and Human Services, Bureau of Laboratories, Lansing, MI 48906, USA

*** Correspondence:**
Corresponding Authors
hansen.zoeann@gmail.com; mannin71@msu.edu

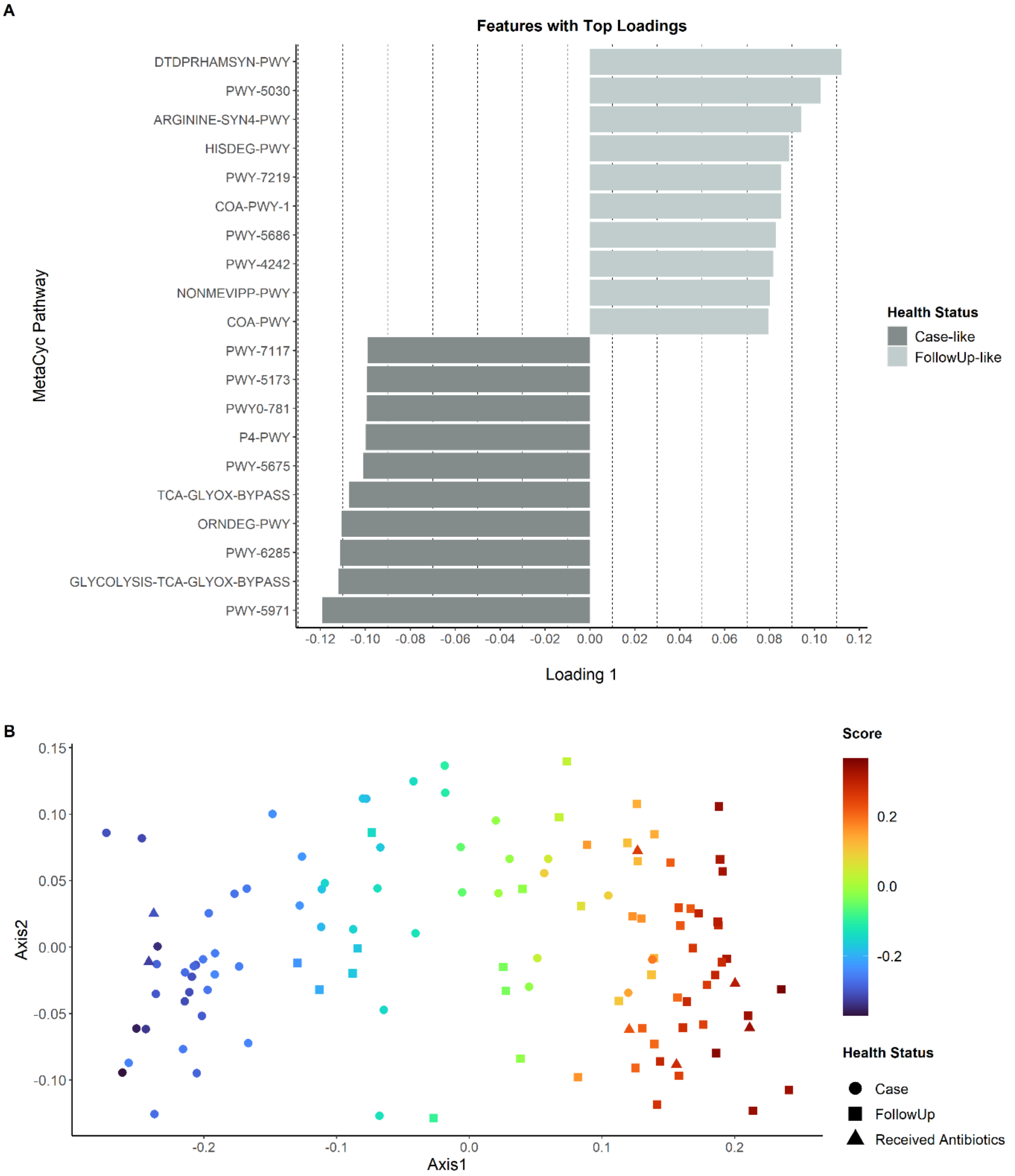

Supplementary Figure S1. Investigation of continuous structure within module compositions reveals metabolic tradeoffs. MMUPHin was used to identify metabolic features contributing to continuous structure among cases and follow-ups. **A)** MetaCyc pathways comprising the top-10 positive and negative consensus loadings of the multidimensional scaling plot are shown; colors were assigned to the loadings based on their "likeness" related to findings from differential abundance analysis (case-like=dark grey; follow-up-like=light grey). **B)** Composition gradients are overlaid on ordination plots based on Bray-Curtis dissimilarity of pathway relative abundances among cases (circles) and follow-ups (squares); individuals reporting use of antibiotics in the two weeks prior to

sample collection are also shown (triangles). The color gradient ("Score") indicates the continuous structure score related to "Loading 1", the top loadings affiliated with the PCA shown in Table S1.

A)

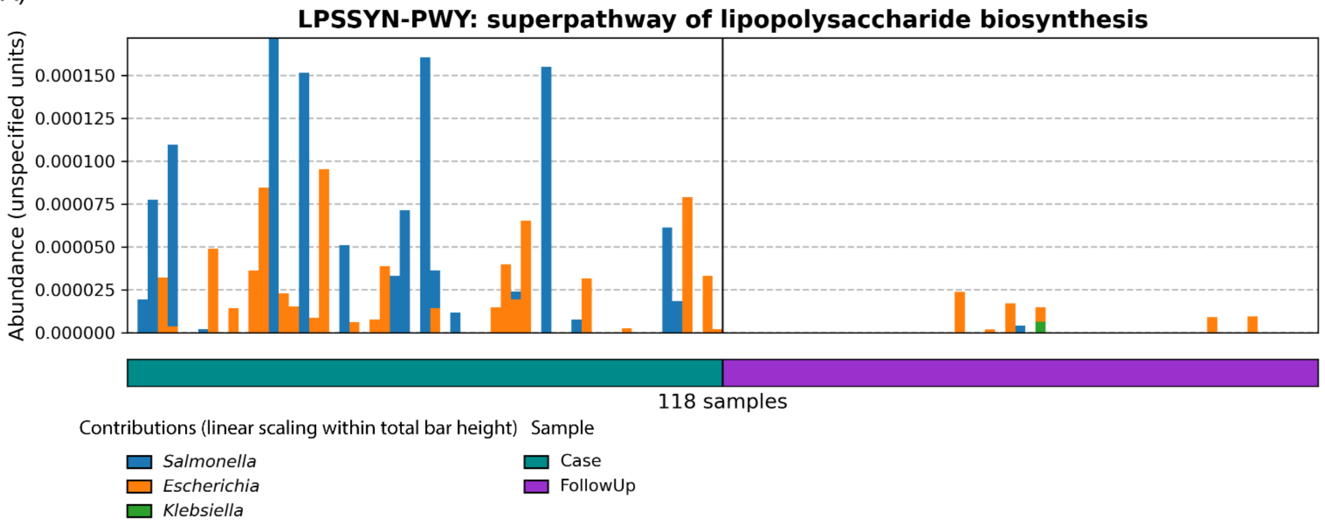

B)

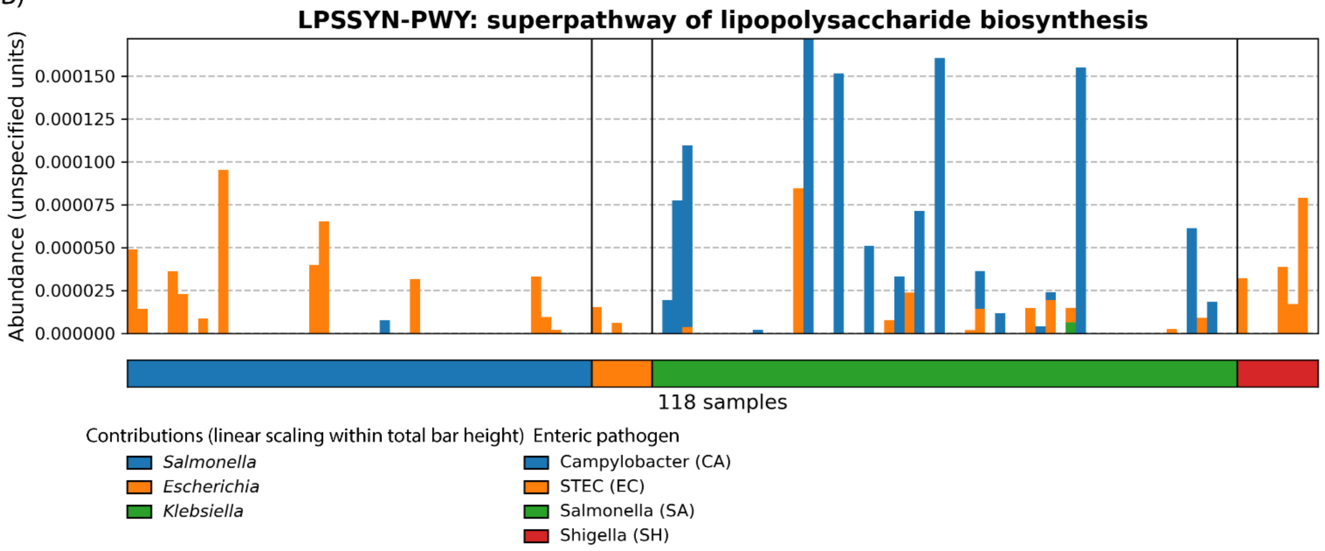

Supplementary Figure S2. Relative abundances of the lipopolysaccharide (LPS) biosynthesis pathway in cases and follow-ups. Barplots show the relative abundance of the LPSSYN-PWY: superpathway of LPS biosynthesis calculated by HUMAnN 3.0 stratified by **A**) health status and **B**) infecting pathogen. The horizontal color bar indicates case (green) vs. follow-up (purple) samples for panel A and *Campylobacter* (blue), *Salmonella* (green), *Shigella* (red), or STEC (orange) in panel B. The 'Contributions' section displays genera associated with the pathway; colors in the stacked barplots show the proportion of relative abundances for LPSSYN-PWY attributed to each genus.

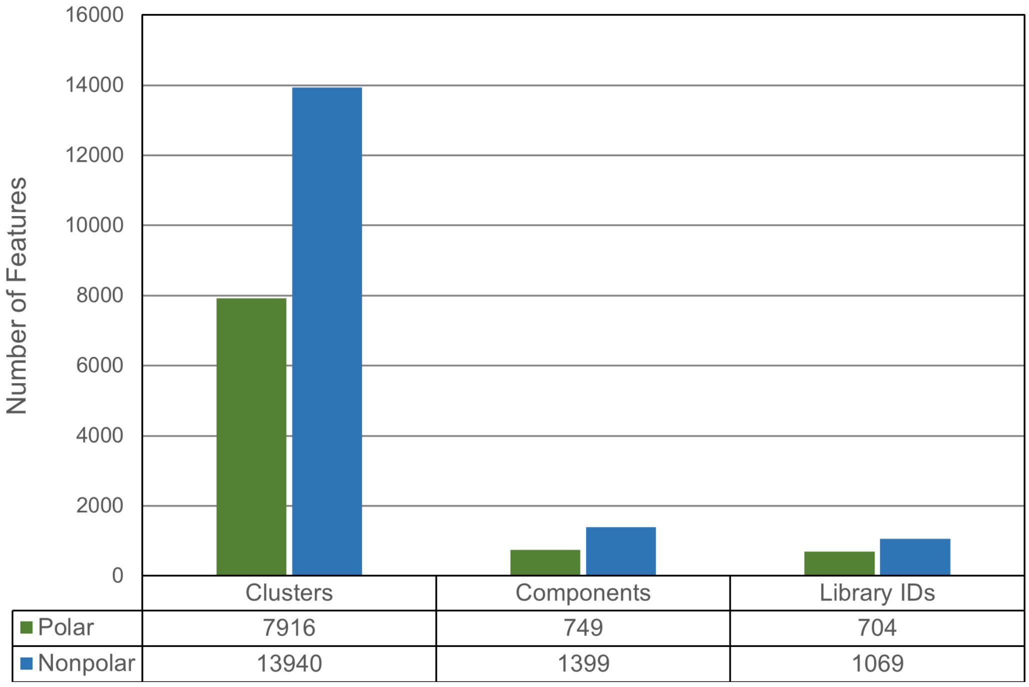

Supplementary Figure S3. Summary of metabolite clusters, network components, and characterized metabolites. A bar graph displaying the quantity of clusters identified by MZmine ("Clusters"), network components determined via Feature Based Molecular Networking (FBMN) ("Components"), and clusters with Library IDs of characterized metabolic compounds determined via GNPS ("Library IDs") for both polar and nonpolar metabolites.

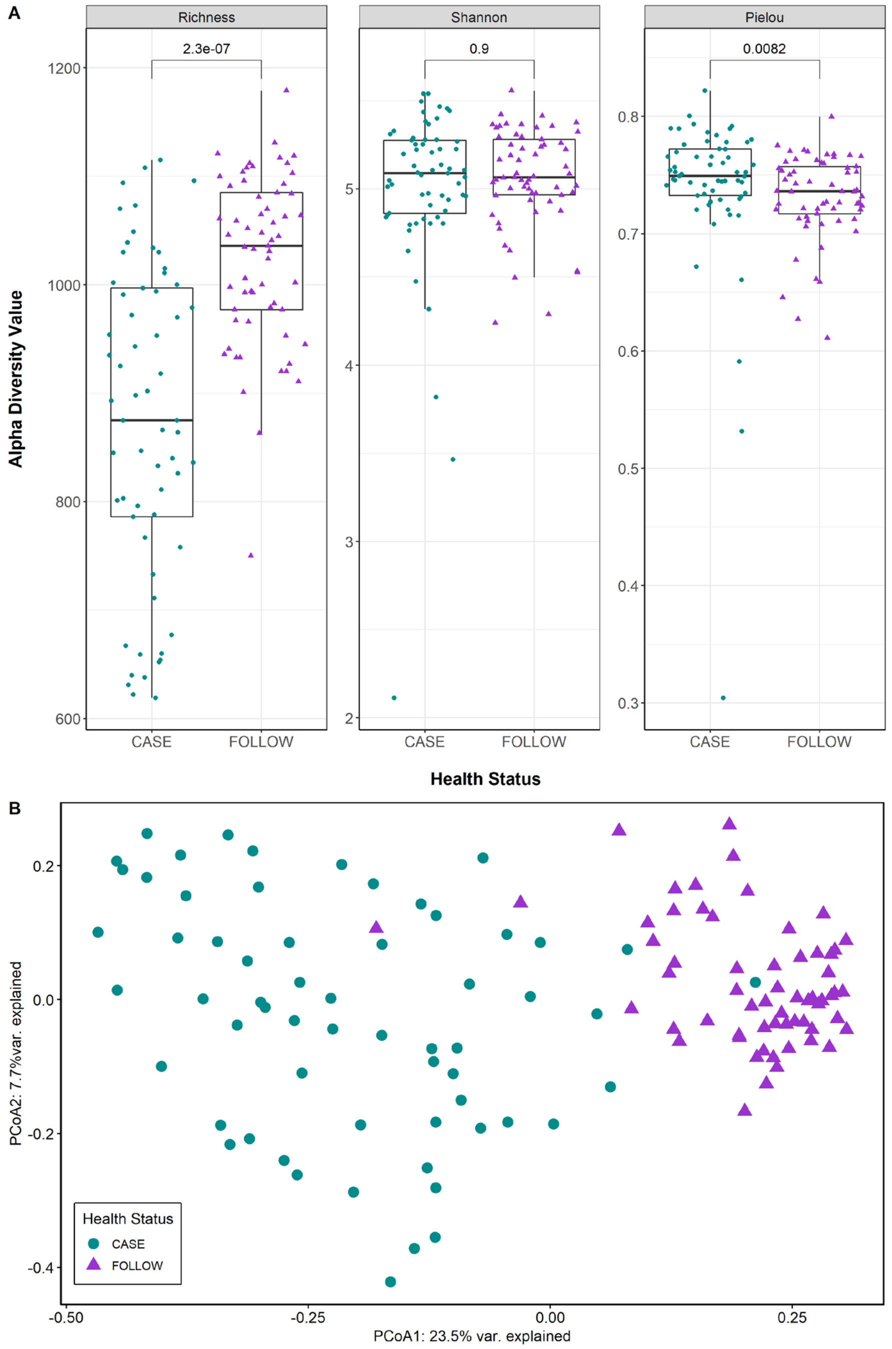

Supplementary Figure S4. Richness and composition of polar metabolites differs among patients during infection and after recovery. **A)** Three measures of alpha diversity (Richness, Shannon Diversity, and Pielou's Evenness) are displayed and stratified by sample for cases (green circles) and follow-ups (purple triangles). Data points are offset from the vertical to allow for clear interpretation of all samples. The median is the thick black bar and the first and third quartiles are shown by the bottom and top of each box, respectively. P-values are shown above each comparison bar and were calculated using the Wilcoxon signed-rank test for paired samples. **B)** Principal coordinates analysis (PCoA) was performed based on Bray-Curtis dissimilarity of polar metabolite quantification for the case and follow-up samples. The first and second coordinate are displayed with their respective percentage of variance explained.

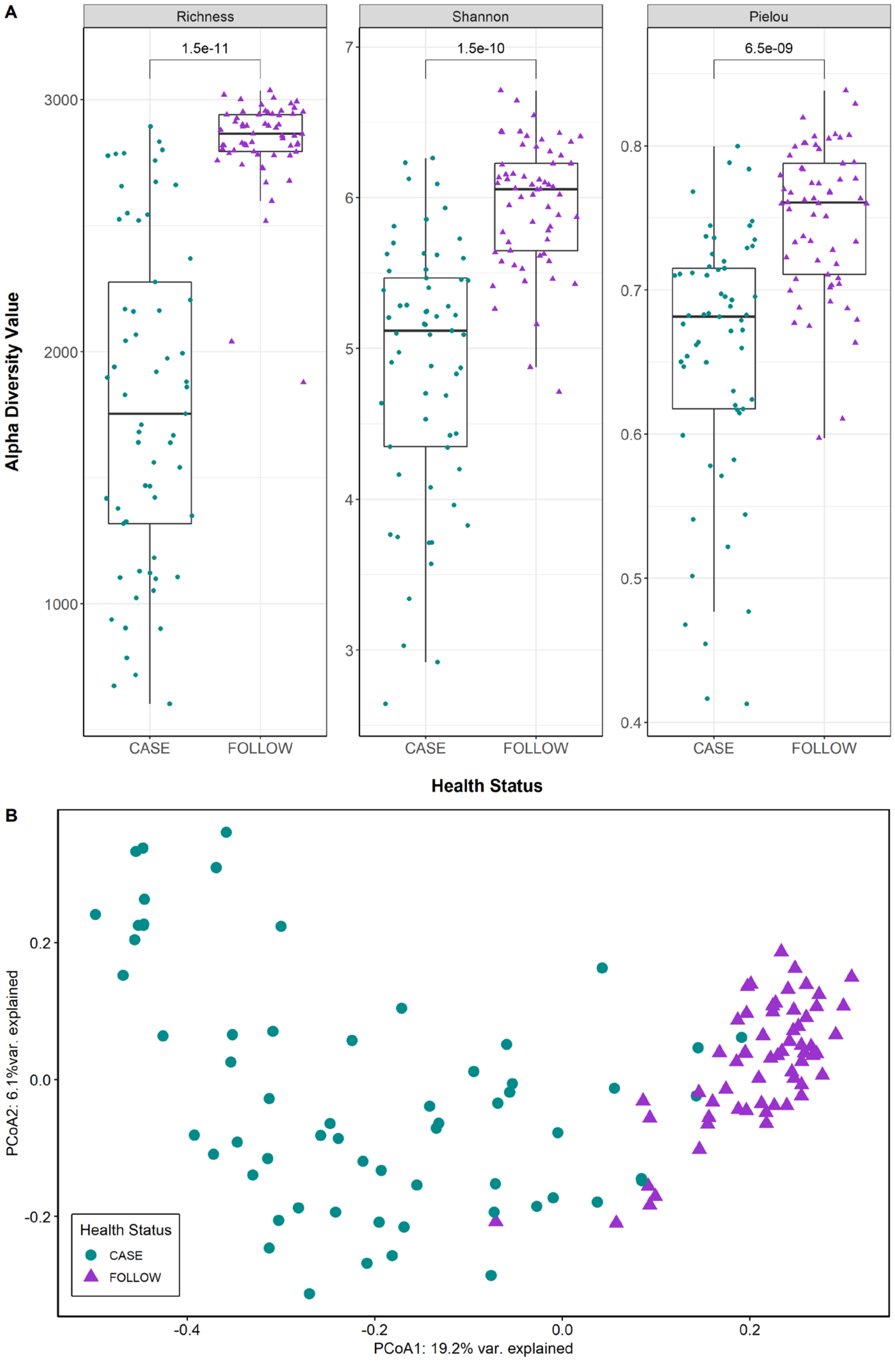

Supplementary Figure S5. Nonpolar metabolite diversity and composition differs between cases and follow-ups. A) Three measures of alpha diversity (Richness, Shannon Diversity, and Pielou's Evenness) are shown for (green circles) and follow-ups (purple triangles). Data points are offset from the vertical to allow for clear interpretation of all samples. The median is shown in each box and the first and third quartiles are at the bottom and top of each box. P-values calculated using the Wilcoxon signed-rank test for paired samples are indicated above the comparison bar. **B)** Principal coordinates analysis (PCoA) based on Bray-Curtis dissimilarity of polar metabolite quantification was also performed for cases and follow-ups. The first and second coordinate are displayed with their respective percentage of variance explained.

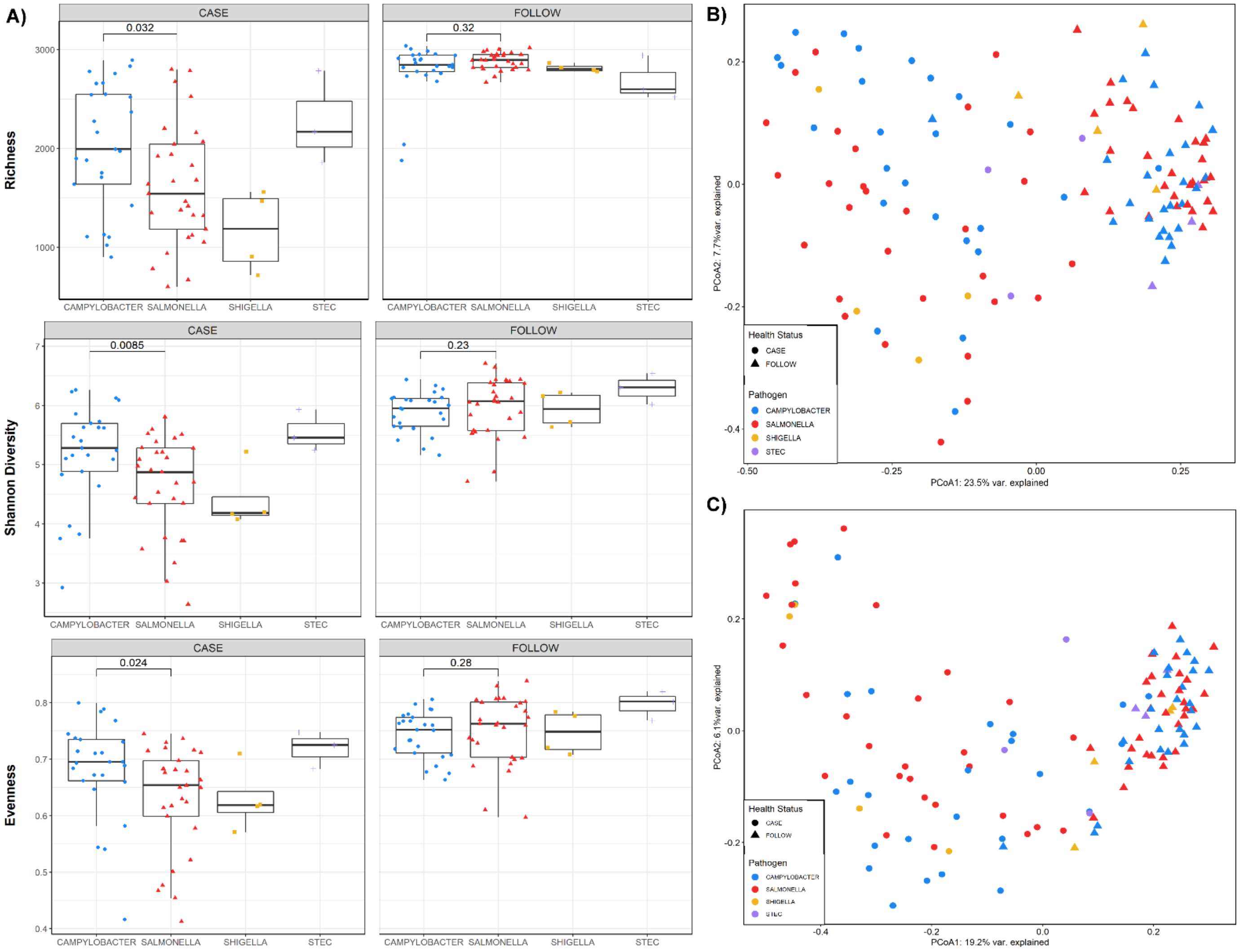

Supplementary Figure S6. Richness and composition of polar and nonpolar metabolites does not appear to be influenced by infecting pathogen. Richness, Shannon Diversity, and Pielou's Evenness are displayed for the **A**) nonpolar metabolites stratified by pathogen indicated on the x-axis and displayed as blue (*Campylobacter*), red (*Salmonella*), yellow (*Shigella*), or violet (STEC). Data points are offset from the vertical. Within each plot, the median is the thick black bar and the first and third quartiles are indicated as the bottom and top of each box. Wilcoxon rank-sum tests were used for cases infected with *Campylobacter* and *Salmonella;* P-values are shown above each comparison bar. Principal coordinates analysis (PCoA) was performed and plotted for cases (circles) and follow-ups (squares) based on Bray-Curtis dissimilarity of **B**) polar and **C**) nonpolar metabolite quantification. Colors refer to the pathogen causing each infection and are the same as those described in panel A. The first and second coordinate are displayed with their respective percentage of variance explained.

A)

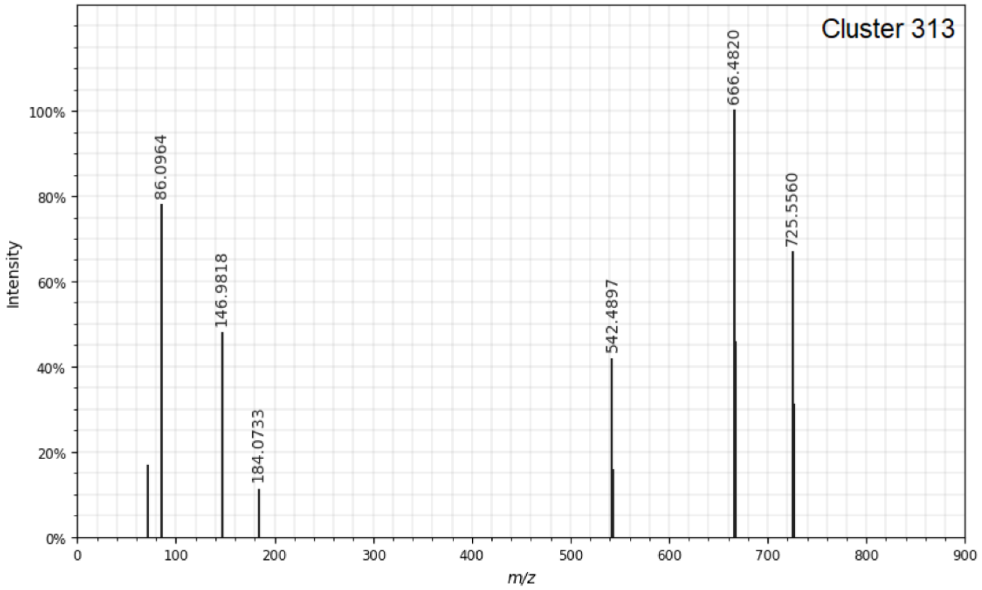

Parent Mass: 725.56
Parent Charge: 1
Sum Precursor Intensity: 4,344.52
RT: 3.92

B)

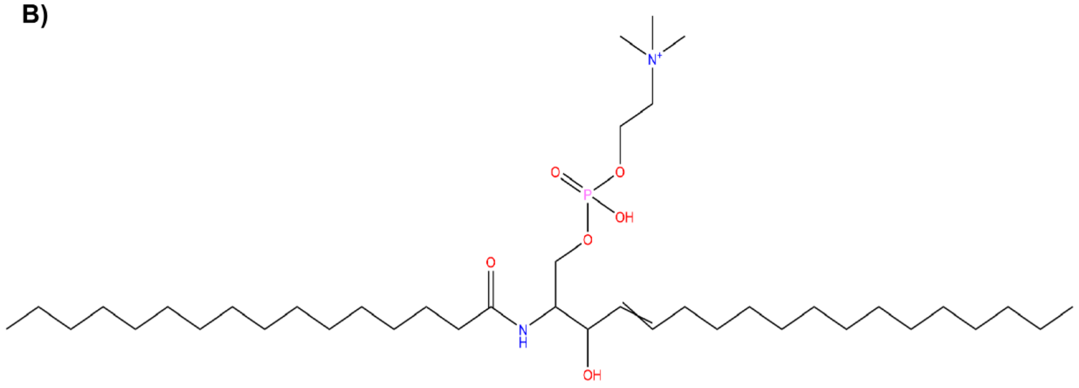

{[2-hexadecanamido-3-hydroxyoctadec-4-en-1-yl]oxy}[2-(trimethylazaniumyl)ethoxy]phosphinic acid

Supplementary Figure S7. Molecular network and MS^2 spectra for Cluster 313, which was abundant in cases. A) The MS^2 spectra Cluster 313 is shown and was successfully annotated as {[2-hexadecanamido-3-hydroxyoctadec-4-en-1-yl]oxy}[2-(trimethylazaniumyl)ethoxy]phosphinic acid. **B**) The chemical structure of Cluster 313 was generated in ChemDraw 20.1.

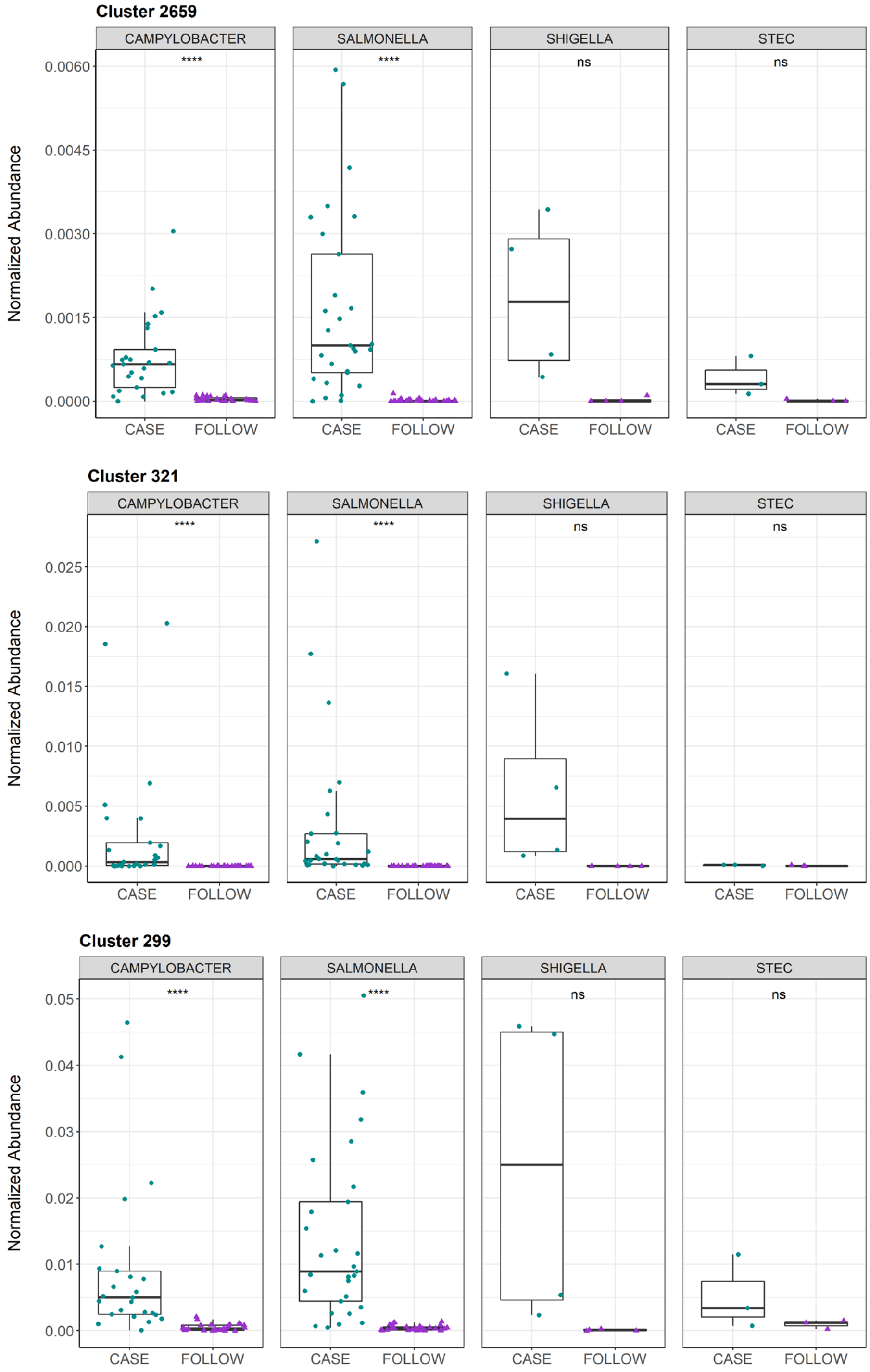

Supplementary Figure S8. Normalized abundance of nonpolar Clusters 2659, 321, and 299 cases and follow-ups by infecting pathogen. Each boxplot per cluster represents the pathogen associated with the case infection, which is stratified by case (green) and follow-up (follow; purple) samples. Data points are offset from the vertical. The median is displayed as a thick black bar in each box; the first and third quartiles are shown by the bottom and the top of each box, respectively. P-values displayed at the top of each plot were calculated using the Wilcoxon signed-rank test. Cutoff levels were: not significant (ns): $p>0.05$, *: $p\leq0.05$, **: $p\leq0.01$, ***: $p\leq0.001$, ****: $p\leq0.0001$.

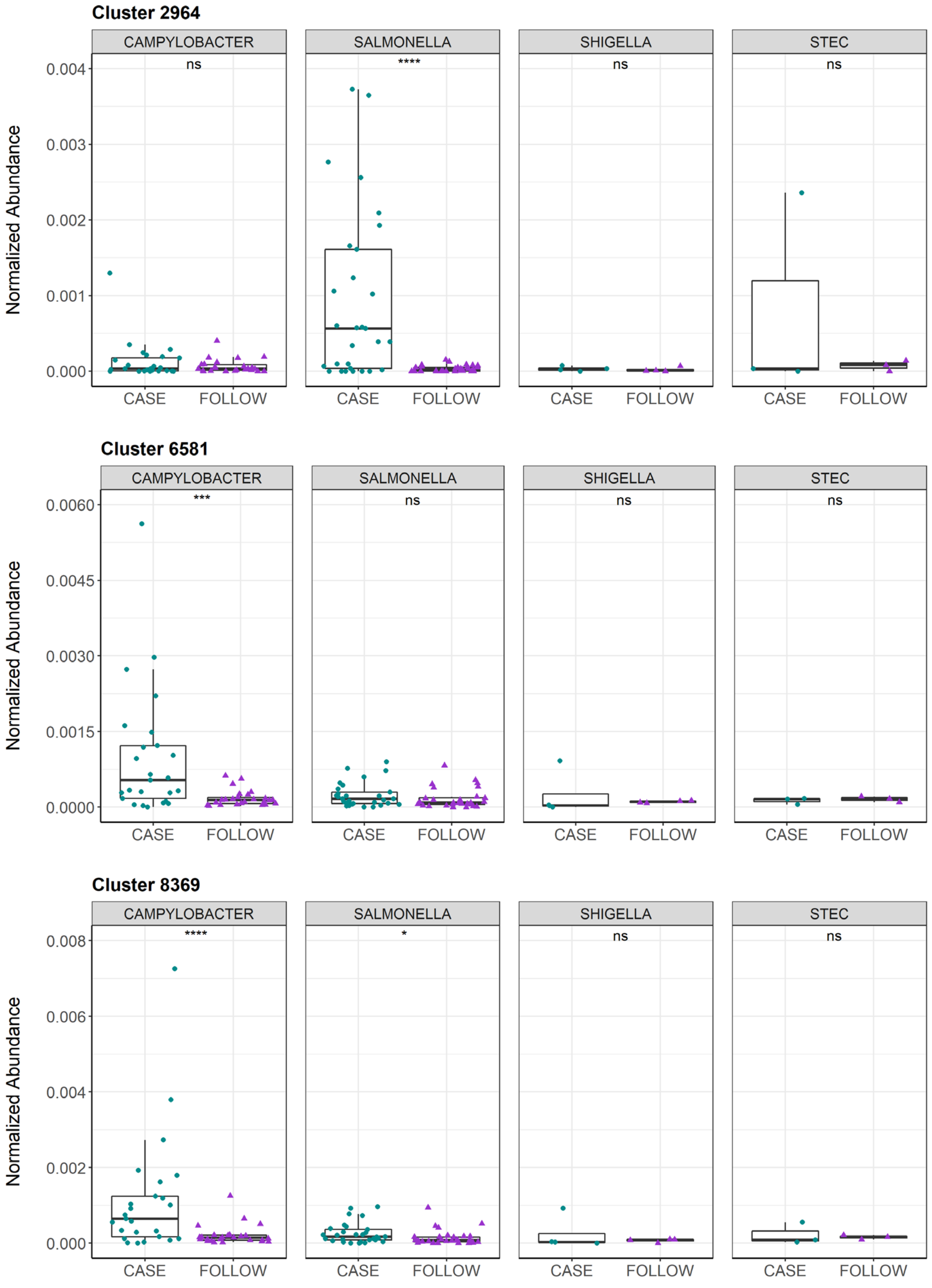

Supplementary Figure S9. Normalized abundances of polar Clusters 2964, 6581, and 8369 across among cases and follow-ups by pathogen. Normalized abundances are displayed, and each boxplot is specific for the infecting pathogen and stratified by sample type; cases are represented by green circles and follow-ups (follow) by purple triangles. Data points are offset from the vertical and the median is represented within each box as a line. The first and third quartiles are indicated by the bottom and the top of each box, respectively. P-values were calculated using the Wilcoxon signed-rank test for paired samples and P-value signifiers are: not significant (ns): p>0.05, *: p≤0.05, **: p≤0.01, ***: p≤0.001, ****: p≤0.0001.

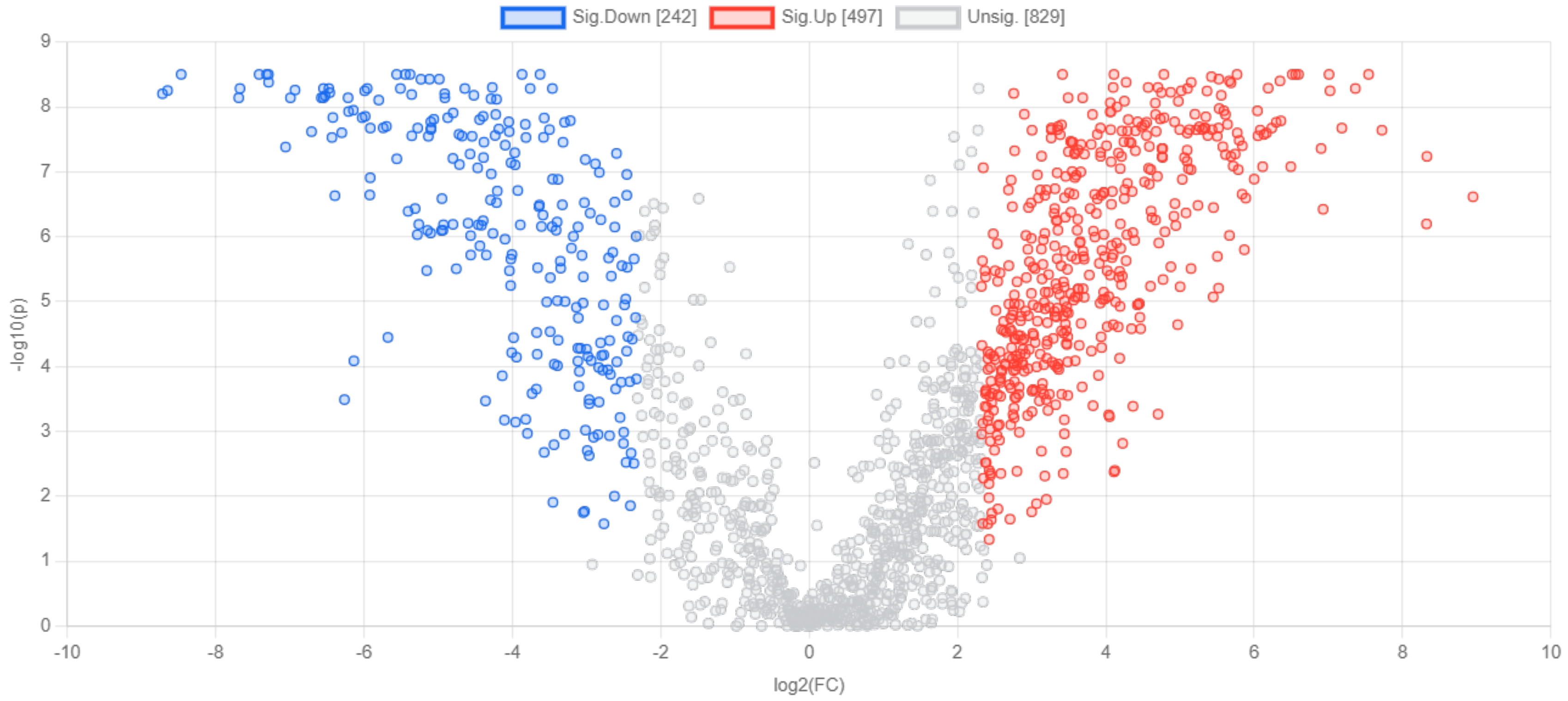

Supplementary Figure S10. Volcano plot demonstrating fold-change of polar metabolites in cases and follow-ups. A fold-change (FC) cutoff of 5.0 and false discovery rate (FDR) threshold of 0.05 were set to identify the strongest signals. The volcano plot shows polar metabolites that were significantly more represented in follow-ups ("Sig.Up", red, positive log_2FC) and in cases ("Sig.Down", blue; negative log_2FC). The x-axis indicates the log_2FC value, whereas the y-axis shows the $-log_{10}(P)$ value. Metabolites that lacked significant associations with either group are shown as gray dots ("Unsig."). The legend at the top of the plot indicates the number of metabolites in each category.

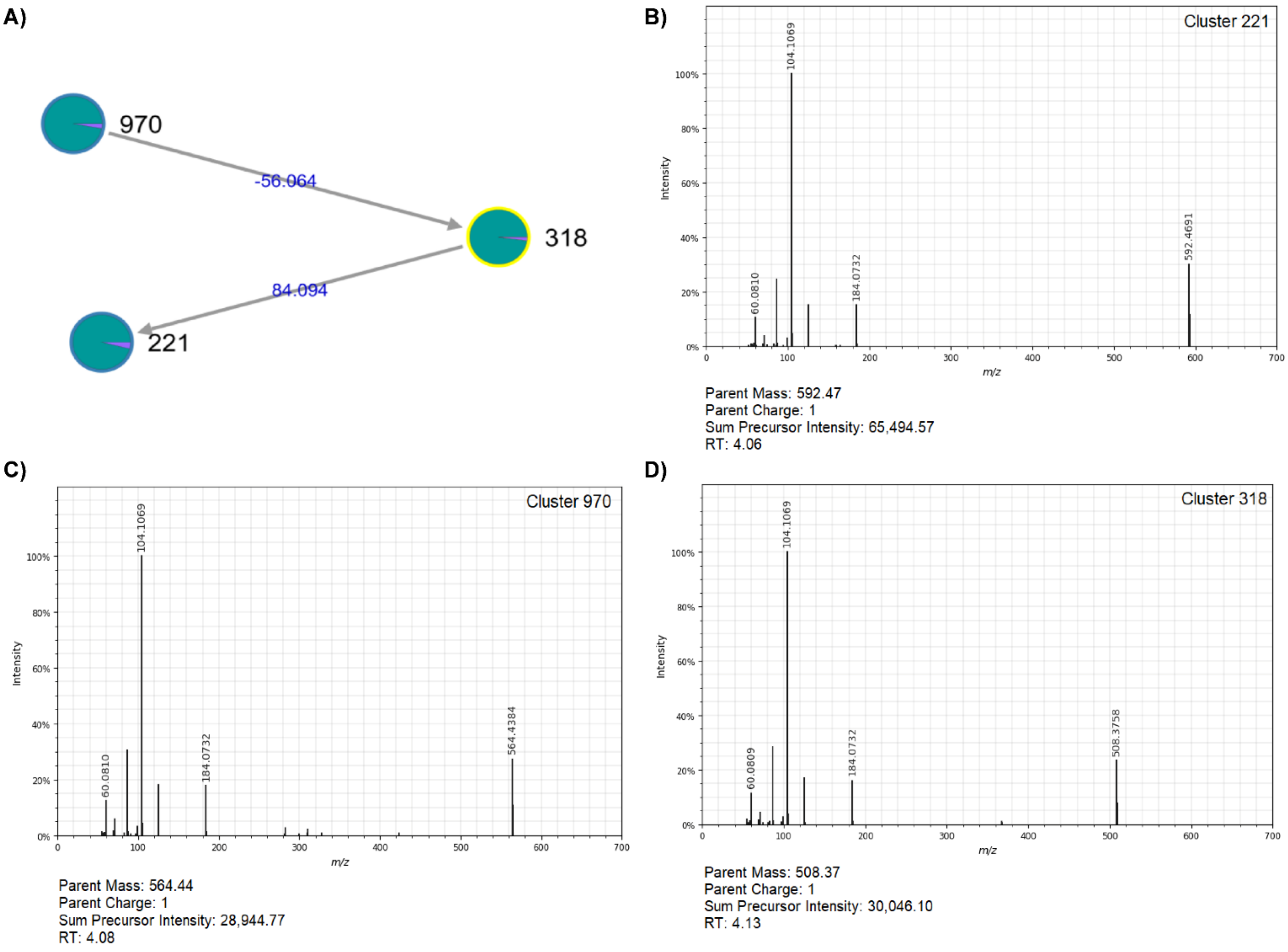

Supplementary Figure S11. Cluster 221 and Cluster 970 were related to known Cluster 318 and found predominantly in case samples. A molecular subnetwork constructed in GNPS (A) shows the interrelatedness of metabolite clusters of interest. Nodes are labeled with their cluster index (black) and edges are labeled with the associated mass difference between two connected nodes (blue). Pie-charts on each node indicate the proportion of that node that was found in cases (green) and follow-ups (purple). The MS^2 spectra for **B**) Cluster 221, **C**) Cluster 970, and **D**) Cluster 318 are shown with peaks of highest intensity labeled with respective masses. Values for parent mass and charge, sum precursor intensity, and retention time (RT) for each cluster are noted below the spectra.

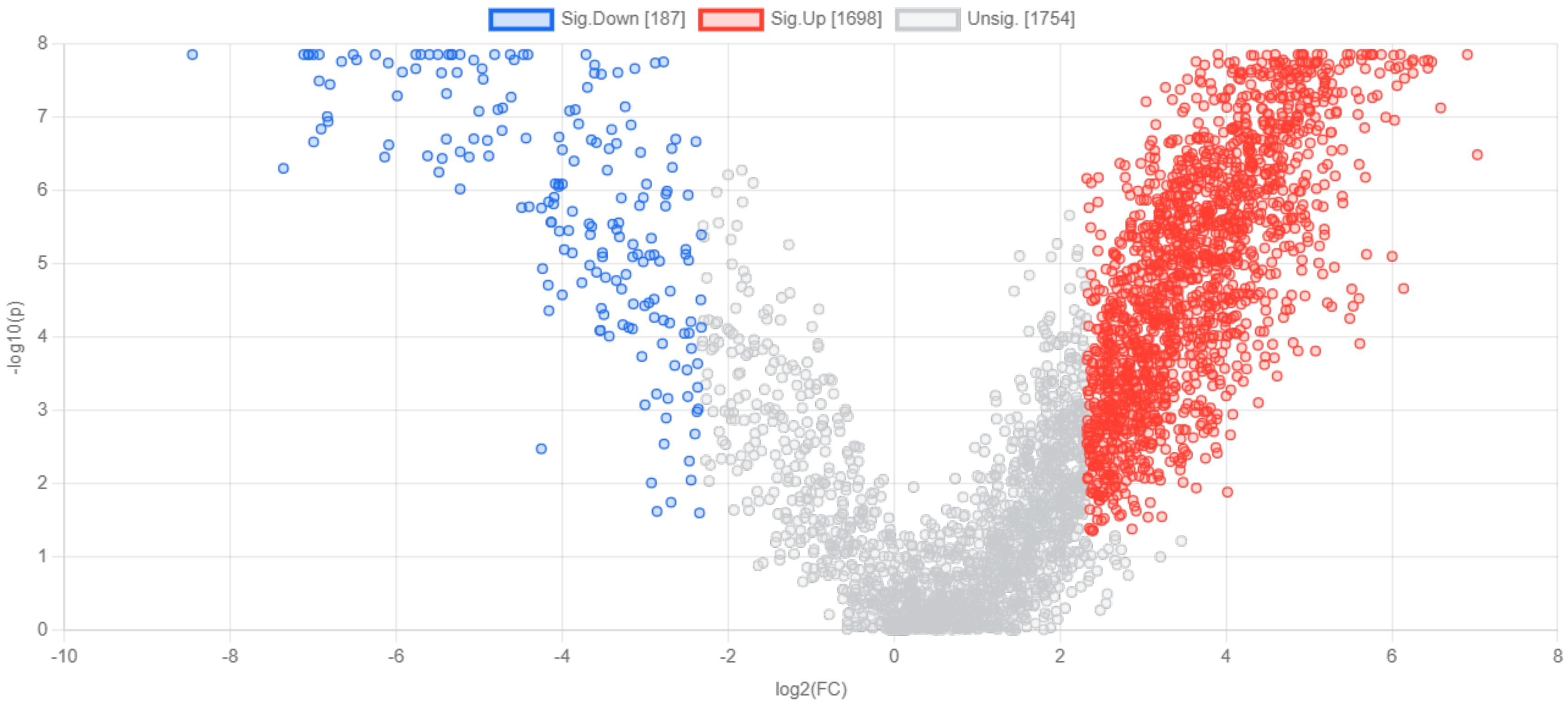

Supplementary Figure S12. Volcano plot demonstrating fold-change of nonpolar metabolites in cases and follow-ups. Fold-change (FC) analysis was performed to explore differentially abundant metabolites among samples. A FC cutoff of 5.0 and FDR threshold of 0.05 were set to identify the strongest signals. The volcano plot shows metabolites that were significantly more represented in follow-ups ("Sig.Up", red, positive log_2FC) and those in cases ("Sig.Down", blue; negative log_2FC).The x-axis indicates the log_2FC value; the y-axis shows the $-log_{10}(P)$ value. Metabolites that lacked significance with these parameter cutoffs are shown as gray dots ("Unsig."). The legend at the top of the plot indicates the number of metabolites in each category.

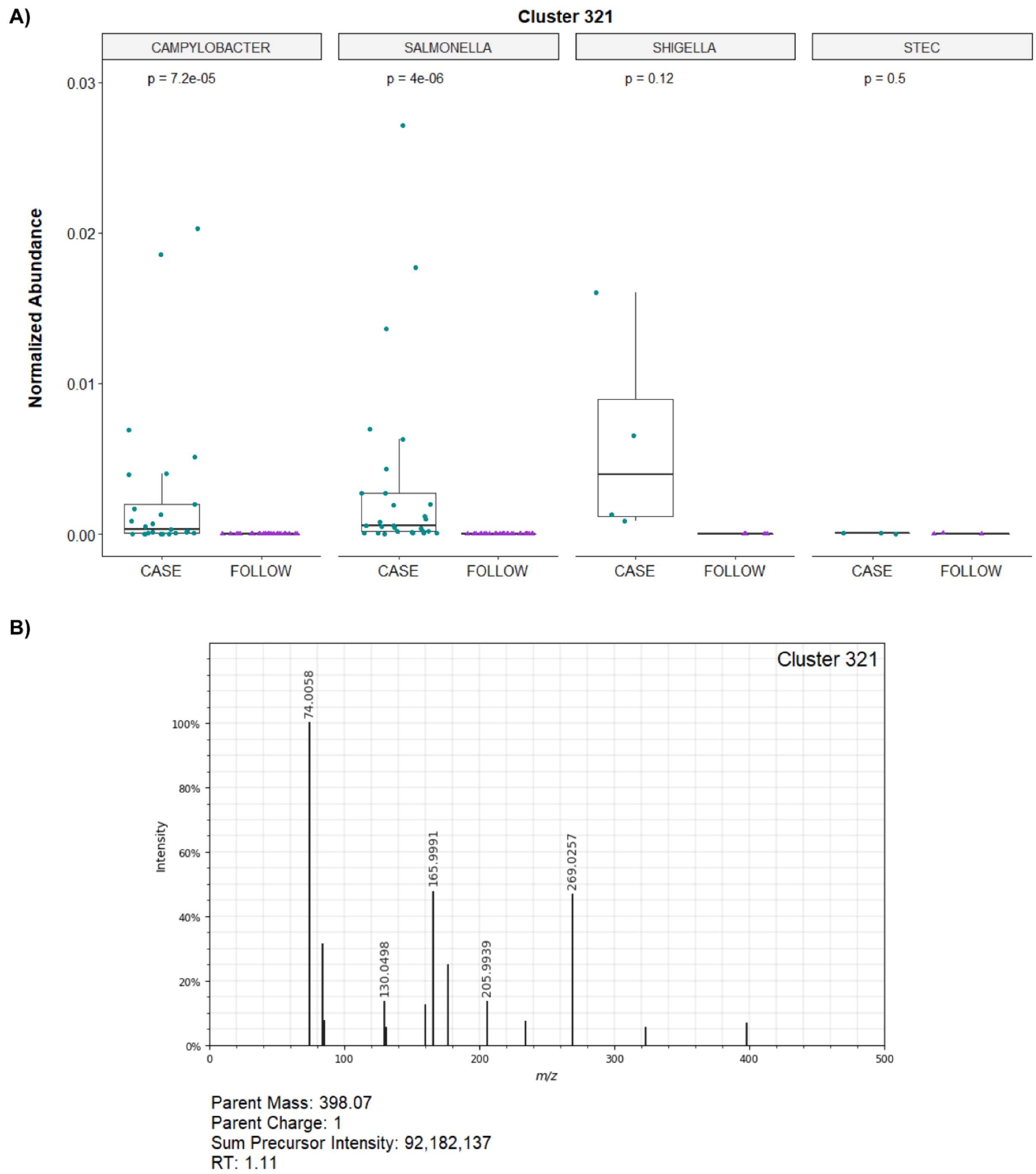

Supplementary Figure S13. Normalized abundance of Cluster 321 among cases and follow-ups separated by infecting pathogen and MS2 spectra. A) Normalized abundances plot is separated by each infecting pathogen and data are shown for cases (green) and follow-ups (purple). Data points are offset from the vertical. Within each box, the median is the thick black bar and the first and third quartiles are at the bottom and the top of each box. Wilcoxon signed-rank test P-values are indicated at the top of the plot for case-follow-up comparisons. **B**) The MS^2 spectra Cluster 321 is shown with peaks of highest intensity labeled with their respective masses.

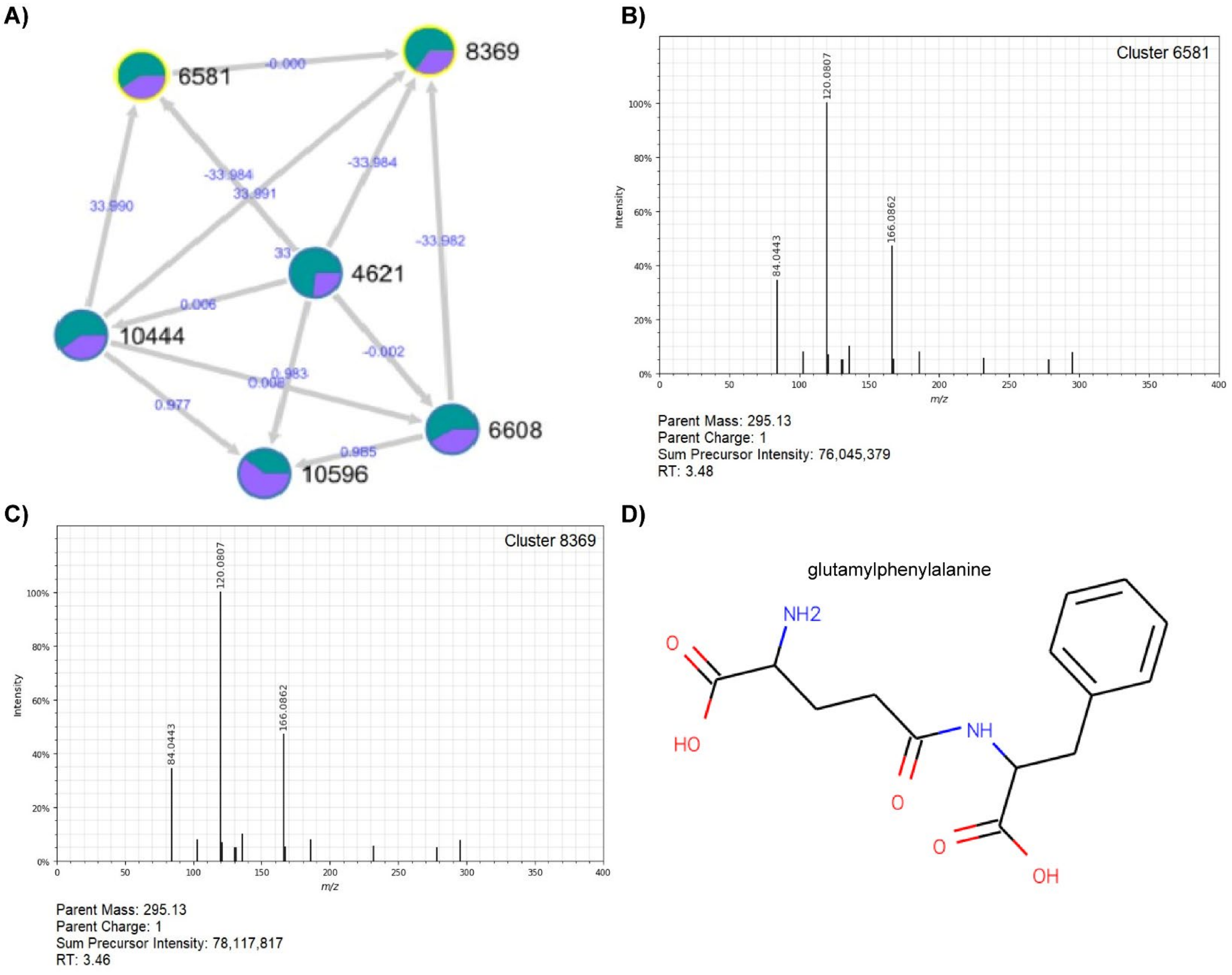

Supplementary Figure S14. Molecular subnetwork and MS^2 spectra for Clusters 6581 and 8369, which were identified as glutamylphenylalanine. A) A molecular subnetwork constructed in GNPS shows the interrelatedness of metabolite clusters 6581 and 8369 with other metabolites. Nodes are labeled with their cluster index (black) and edges are labeled with the associated mass difference between two connected nodes (blue). Pie-charts on each node indicate the proportion of that node that was found in cases (green) and follow-ups (purple). The MS^2 spectra for Cluster 6581 (**B**) and Cluster 8369 (**C**) are shown. **D**) Both clusters were annotated as an isomer of glutamylphenylalanine; the chemical structure was generated in ChemDraw 20.1.

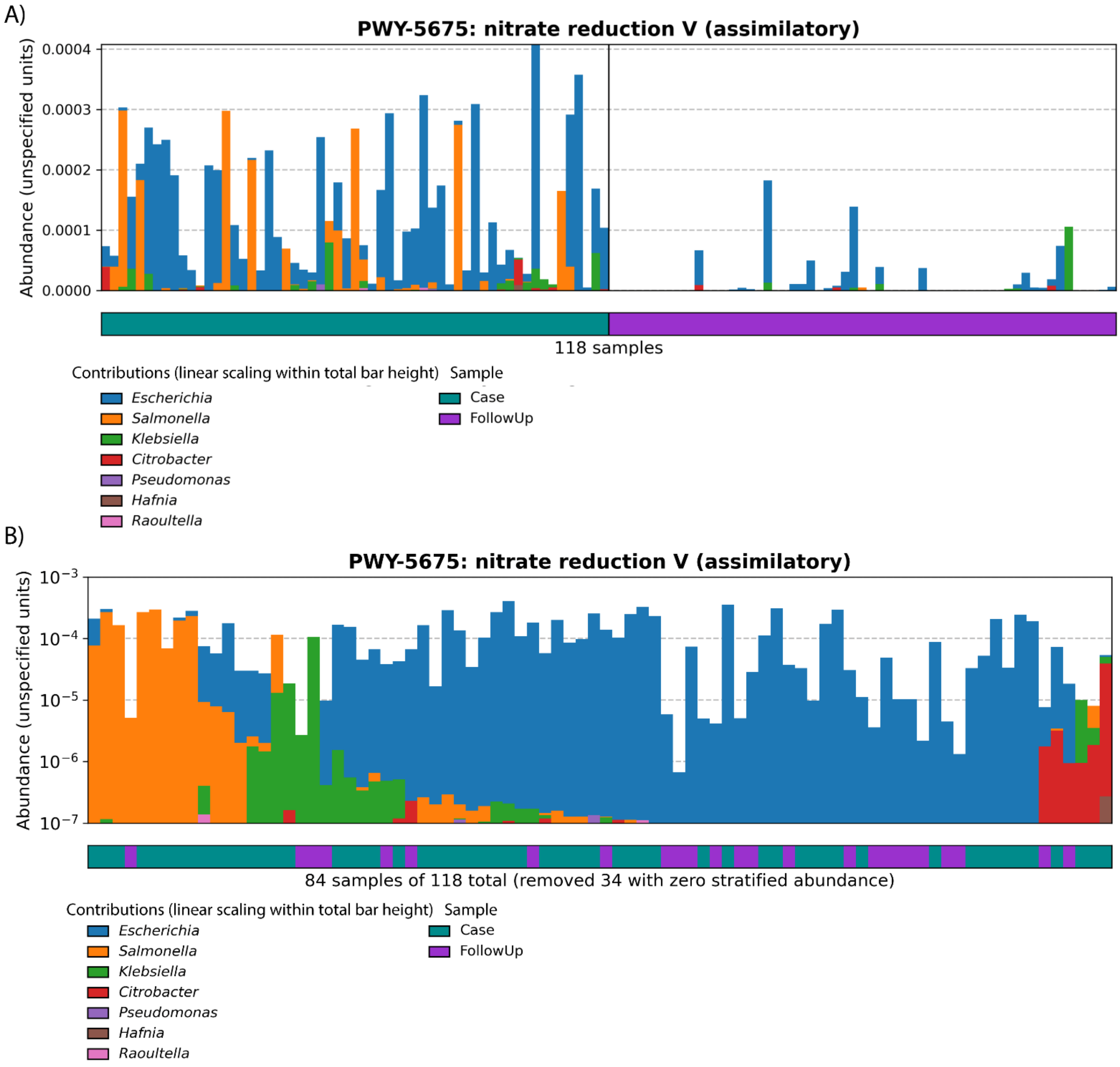

Supplementary Figure S15. Relative abundances of PWY-5675: nitrate reduction V among infected and recovered patients. A) Barplots show the relative abundance of PWY-5675 calculated by HUMAnN 3.0 stratified by health status. **B**) Sample relative abundances were also clustered by Bray-Curtis dissimilarity to explore clustering relevant to specific genera associations and abundance. The horizontal color bars on the bottom of each plot designate case (green) vs. follow-up (purple) samples. The ‘Contributions’ section displays genera found to be associated with the pathway of interest; colors in the stacked barplots show the proportion of relative abundances for each pathway attributed to that specific genus.

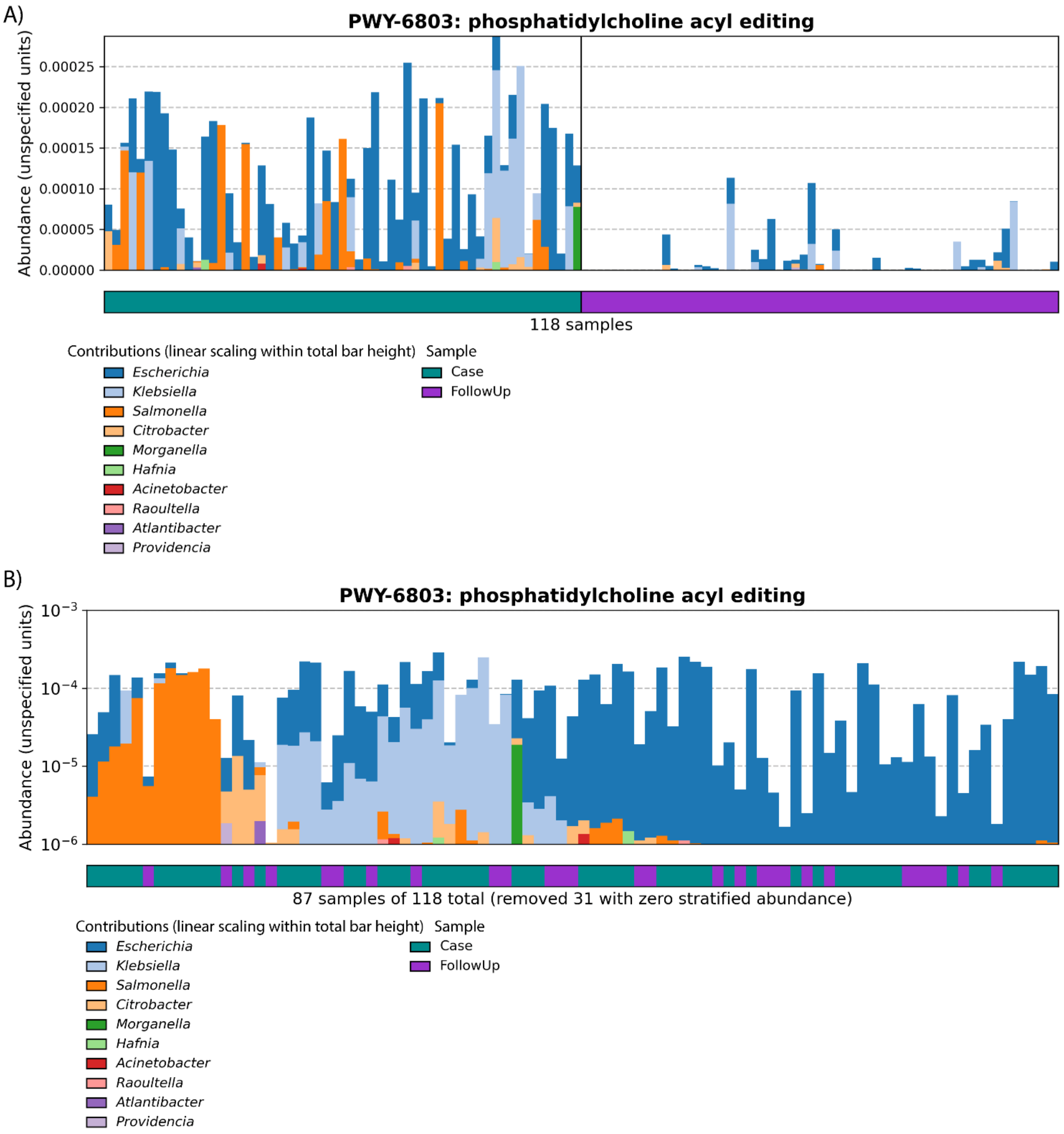

Supplementary Figure S16. Relative abundances of PWY-6803: phosphatidylcholine acyl editing among infected and recovered patients. (A) Barplots show the relative abundance of PWY-6803 calculated by HUMAnN 3.0 stratified by health status. (**B**) Sample relative abundances were also clustered by Bray-Curtis dissimilarity to explore clustering relevant to specific genera associations and abundance. The horizontal color bars on the bottom of each plot designate case (green) vs. follow-up (purple) samples. The 'Contributions' section displays genera found to be associated with the pathway of interest; colors in the stacked barplots show the proportion of relative abundances for each pathway attributed to that specific genus.
